# Supplementary material for: Vascularized human cortical organoids (vOrganoids) model cortical development in vivo
Source: PLoS Biol. 2020 May 13;18(5):e3000705. doi: 10.1371/journal.pbio.3000705 (PMC7250475; doi:10.1371/journal.pbio.3000705)
Supplement: S1 Checklist — MDAR, Materials Design Analysis Reporting. (DOCX) [file pbio.3000705.s001.docx]

Materials Design Analysis Reporting (MDAR)

Checklist for Authors

The MDAR framework establishes a minimum set of requirements in transparent reporting applicable to studies in the life sciences (see Statement of Task: doi:10.31222/osf.io/9sm4x.). The MDAR checklist is a tool for authors, editors and others seeking to adopt the MDAR framework for transparent reporting in manuscripts and other outputs. Please refer to the MDAR Elaboration Document for additional context for the MDAR framework.

Materials

| **Antibodies** | **Yes (indicate where provided: page no/section/legend)** | **n/a** |
| --- | --- | --- |
| For commercial reagents, provide supplier name, catalogue number and RRID, if available. | Yes ( page27-28**/** Materials and Methods) |  |
|  |  |  |
| **Cell materials** | **Yes (indicate where provided: page no/section/legend)** | **n/a** |
| **Cell lines:** Provide species information, strain. Provide accession number in repository **OR** supplier name, catalog number, clone number, **OR** RRID | Yes ( page26-27/ Materials and Methods/3D vOrganoids culture and differentiation procedure) |  |
| **Primary cultures:** Provide species, strain, sex of origin, genetic modification status. |  | n/a |
|  |  |  |
| **Experimental animals** | **Yes (indicate where provided: page no/section/legend)** | **n/a** |
| **Laboratory animals:** Provide species, strain, sex, age, genetic modification status. Provide accession number in repository **OR** supplier name, catalog number, clone number, **OR** RRID | Yes ( page25/ Materials and Methods /Mice) |  |
| **Animal observed in or captured from the field:** Provide species, sex and age where possible |  | n/a |
| **Model organisms:** Provide Accession number in repository (where relevant) **OR** RRID |  | n/a |
|  |  |  |
| **Plants and microbes** | **Yes (indicate where provided: page no/section/legend)** | **n/a** |
| **Plants:** provide species and strain, unique accession number if available, and source (including location for collected wild specimens) |  | n/a |
| **Microbes:** provide species and strain, unique accession number if available, and source |  | n/a |
|  |  |  |
| **Human research participants** | **Yes (indicate where provided: page no/section/legend)** | **n/a** |
| Identify authority granting ethics approval (IRB or equivalent committee(s), provide reference number for approval. | Yes ( page25/ Materials and Methods / Ethics Statement) |  |
| Provide statement confirming informed consent obtained from study participants. | Yes (page25/ Materials and Methods / Ethics Statement) |  |
| Report on age and sex for all study participants. |  | n/a |

Design

| **Study protocol** | **Yes (indicate where provided: page no/section/legend)** | **n/a** |
| --- | --- | --- |
| For clinical trials, provide the trial registration number **OR** cite DOI in manuscript. |  | n/a |
|  |  |  |
| **Laboratory protocol** | **Yes (indicate where provided: page no/section/legend)** | **n/a** |
| Provide DOI or other citation details if detailed step-by-step protocols are available. |  | n/a |
|  |  |  |
| **Experimental study design (statistics details)** | **Yes (indicate where provided: page no/section/legend)** | **n/a** |
| State whether and how the following have been done**, or** if they were not carried out. |  |  |
| Sample size determination | Yes (page 7/ Results) |  |
| Randomisation |  | n/a |
| Blinding |  | n/a |
| Inclusion/exclusion criteria |  | n/a |
|  |  |  |
| Sample definition and in-laboratory replication | **Yes (indicate where provided: page no/section/legend)** | **n/a** |
| State number of times the experiment was replicated in laboratory | Yes (page 5-45/Figure legend, Supporting information) |  |
| Define whether data describe technical or biological replicates | Yes (page 5-35/Figure legend, Supporting information) |  |
|  |  |  |
| Ethics | **Yes (indicate where provided: page no/section/legend)** | **n/a** |
| Studies involving human participants: State details of authority granting ethics approval (IRB or equivalent committee(s), provide reference number for approval. | Yes (page25/ Materials and Methods / Ethics Statement) |  |
| Studies involving experimental animals: State details of authority granting ethics approval (IRB or equivalent committee(s), provide reference number for approval. | Yes (page25/ Materials and Methods / Ethics Statement) |  |
| Studies involving specimen and field samples: State if relevant permits obtained, provide details of authority approving study; if none were required, explain why. |  | n/a |
|  |  |  |
| Dual Use Research of Concern (DURC) | **Yes (indicate where provided: page no/section/legend)** | **n/a** |
| If study is subject to dual use research of concern, state the authority granting approval and reference number for the regulatory approval |  | n/a |

Analysis

| **Attrition** | **Yes (indicate where provided: page no/section/legend)** | **n/a** |
| --- | --- | --- |
| State if sample or data point from the analysis is excluded, and whether the criteria for exclusion were determined and specified in advance. | Yes (page 31/Materials and Methods/ Single-cell RNA-seq data pre-processing) |  |
|  |  |  |
| **Statistics** | **Yes (indicate where provided: page no/section/legend)** | **n/a** |
| Describe statistical tests used and justify choice of tests. | Yes (page 34/ Materials and Methods / Quantification and Statistical analysis) |  |
|  |  |  |
| **Data Availability** | **Yes (indicate where provided: page no/section/legend)** | **n/a** |
| State whether newly created datasets are available, including protocols for access or restriction on access. | Yes. All relevant data are within the paper and its Supporting Information files. The accession number for the RNA sequencing data reported in this paper is GEO: GSE131094.  (page 34/ Materials and Methods/ Data Resources/ GEO:GSE131094) |  |
| If data are publicly available, provide accession number in repository or DOI or URL. |  | n/a |
| If publicly available data are reused, provide accession number in repository or DOI or URL, where possible. | Yes (reference as: Nowakowski, T.J. et al., 2017) |  |
|  |  |  |
| **Code Availability** | **Yes (indicate where provided: page no/section/legend)** | **n/a** |
| For all newly generated code and software essential for replicating the main findings of the study: |  |  |
| State whether the code or software is available. | Yes (page 30-34/ Materials and Methods) |  |
| If code is publicly available, provide accession number in repository, or DOI or URL. |  | n/a |

Reporting

| **Adherence to community standards** | **Yes (indicate where provided: page no/section/legend)** | **n/a** |
| --- | --- | --- |
| MDAR framework recommends adoption of discipline-specific guidelines, established and endorsed through community initiatives. Journals have their own policy about requiring specific guidelines and recommendations to complement MDAR. |  |  |
| State if relevant guidelines (eg., ICMJE, MIBBI, ARRIVE) have been followed, and whether a checklist (eg., CONSORT, PRISMA, ARRIVE) is provided with the manuscript. | All authors have read, and confirm that they meet, ICMJE criteria for authorship. |  |
