# Supplementary figures and images for: Vascularized human cortical organoids (vOrganoids) model cortical development in vivo
Source: PLoS Biol. 2020 May 13;18(5):e3000705. doi: 10.1371/journal.pbio.3000705 (PMC7250475; doi:10.1371/journal.pbio.3000705)

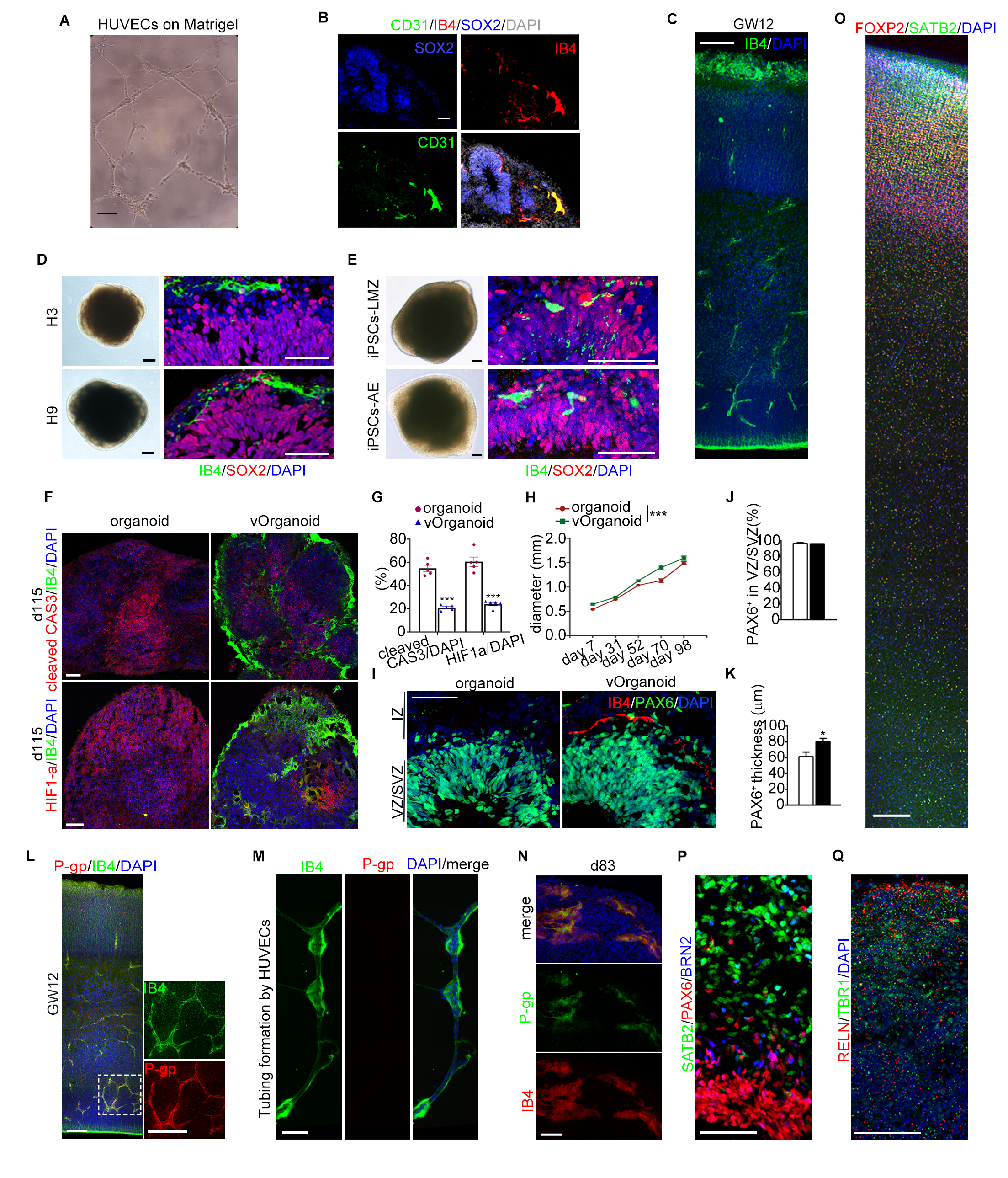

Supplement: S1 Fig — (A) Representative figure showing the tube formation of HUVECs on matrigel. Scale bar, 200 μm. (B) Representative immunofluorescence staining figure for CD31, IB4, and SOX2 to illustrate that tube-like vascular systems were formed in vOrganoids at 45 days. Scale bar, 50 μm. (C) Representative immunostaining figure for IB4 in the human cortical cryosections at GW12 to show the distribution of blood vessels in the human cortex. Scale bar, 100 μm. (D) Representative BF and immunohistochemical images of vOrganoids derived from hESC-3 and hESC-9 lines. BF images showed on the left and immunohistochemical images for vessel (IB4, green) and progenitor (SOX2, red) showed on the right. Scale bar, 100 μm (left panels), 50 μm (right panels). (E) Representative BF and immunohistochemical images of vOrganoids derived from iPSCs-AE and iPSCs-LMZ cell lines. BF images showed on the left and immunohistochemical images for vessel (IB4, green) and progenitor (SOX2, red) showed on the right. Scale bar, 100 μm (left panels), 50 μm (right panels). (F) Representative immunofluorescence staining figure for cleaved CASPASE 3, IB4 (upper) and HIF1α, IB4 (lower) in the control nonvascularized organoids and vOrganoids at d115. Scale bar, 500 μm. (G) Quantification of the percentages of cleaved CASPASE 3+ cells (left) and HIF1α+ cells (right) within all cells (DAPI+) in the control organoids and vOrganoids at d115, respectively. For cleaved CASPASE 3, n = 5, 5 slices of the control organoids and vOrganoids from three independent experiments. For HIF1α, n = 5, 5 slices of control and vOrganoids in three independent experiments. Data are represented as mean ± SEM, independent samples t test, ***p < 0.001. (H) The diameters of organoids and vOrganoids generated from H9 at day 7, day 31, day 52, day 70 and day 98, respectively. n = 11, 11, 11, 11, 11 for day 7, day 31, day 52, d 70, and day 98, respectively. Data are represented as mean ± SEM, two-way ANOVA analysis, ***p < 0.001. (I) Representati [file pbio.3000705.s002.tif]

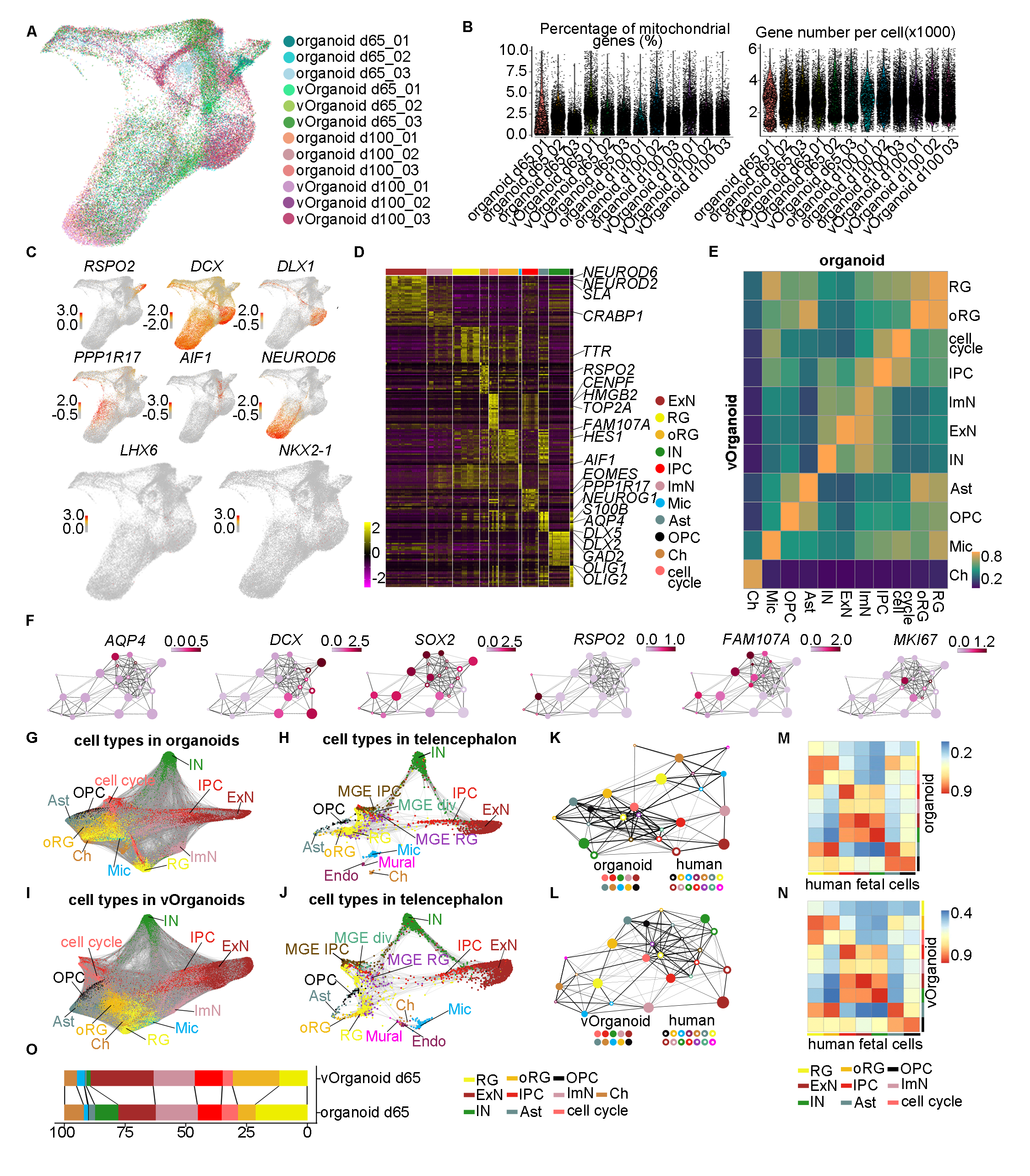

Supplement: S2 Fig — (A) The cell distributions of each sample of control organoid and vOrganoid were showed in the UMAP plots. As for the control organoids and vOrganoids at each time point, three independent batches of experiments were performed. And in total, 12 samples were included in the studies. Each sample was colored differently in the UMAP plot. (B) Quality control for samples: each dot represents a single cell. Cells with mitochondrial gene percentage >10% (left panel), as well as gene number per cell (nGene) <200 and >6,000 (right panel), were discard in the following analysis. (C) The expression of known gene markers was visualized by UMAP plots and was colored by the expression level (red, high; gray, low). (D) Heatmap showing the expression of DEGs across clusters. Some canonical marker genes were labeled. (E) The transcriptomic correlations between the cell types of organoids and vOrganoid were visualized via heatmap (correlation coefficient: yellow, high; purple, low). (F) The expression of the well-known gene markers in different cell types is showed in the PAGA plots. AQP4, DCX, SOX2, RSPO2, FAM107A, and MKI67 are the specific genes for astrocytes, immature neurons, RG/oRG, choroid plexus, oRG, and cell cycle active cells, respectively. Nodes are colored according to the gene expression levels (light pink, low; dark red, high). (G-H) After being integrated, the cell distributions of organoids (G) and human fetal telencephalon (H) were visualized in a forced-directed graph separately. Each dot represents a single cell and is colored according to the cell types. The width of edges is scaled with the cell–cell connectivity. (I-J) After being integrated, the cell distributions of vOrganoids (I) and human fetal telencephalon (J) were visualized in a forced-directed graph separately. Each dot represents a single cell and is colored according to the cell types. The width of edges is scaled with the cell–cell connectivity. (K-L) The correlations between the cell types of orga [file pbio.3000705.s003.tif]

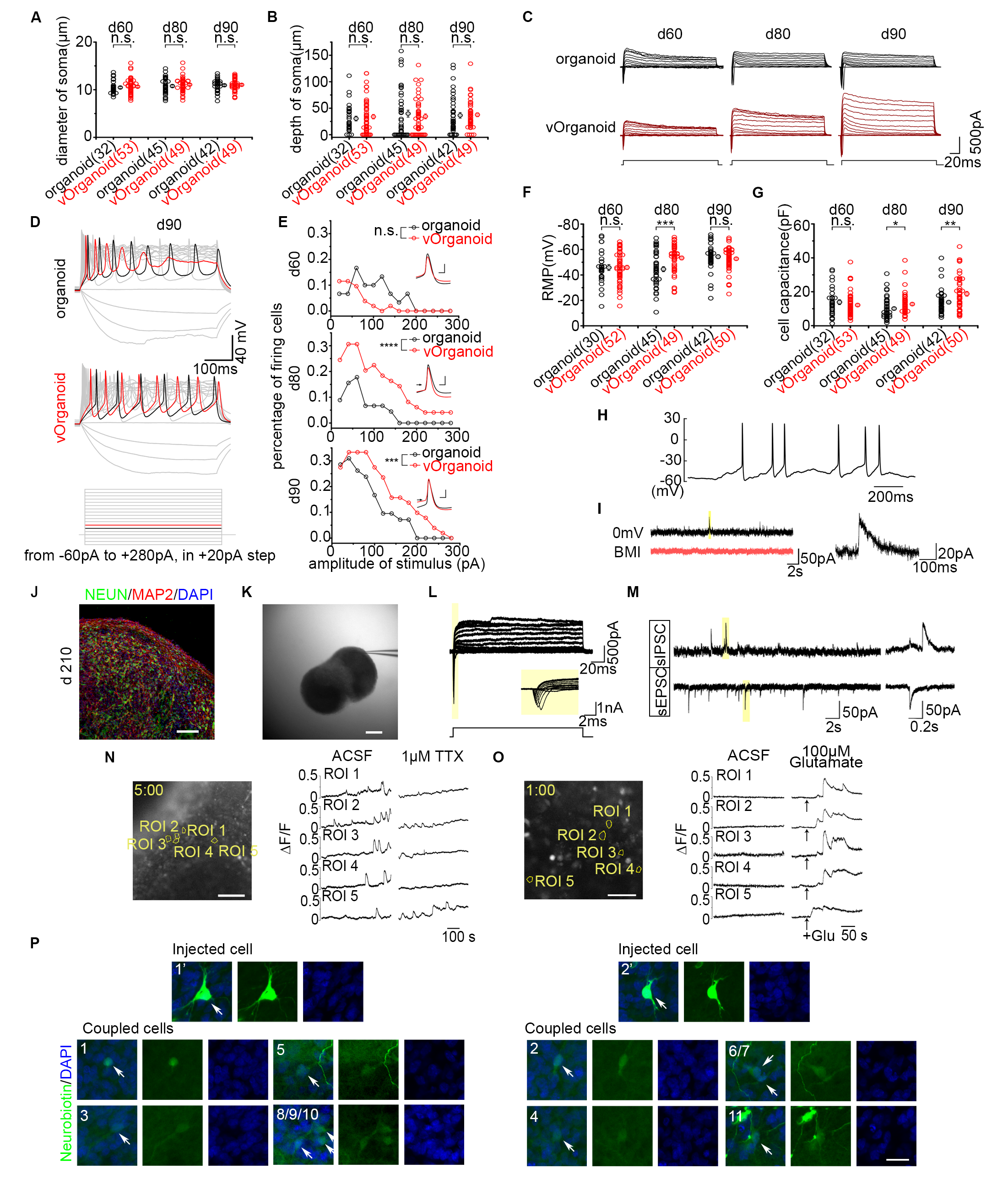

Supplement: S3 Fig — (A) Diameters of somata of recorded cells from control organoids and vOrganoids at day 60, day 80, and day 90. Open circles indicate data from individual cells. Filled circles indicate the mean value. Data are represented as mean ± SEM, p = 0.6026, 0.3494, and 0.7491 for day 60, day 80, and day 90, two-sample t test, *p < 0.05, **p < 0.01, ***p < 0.001. (B) Depth of somata of recorded cells from organoids and vOrganoids at day 60, day 80, and day 90. Open circles indicate data from individual cells. Filled circles indicate the mean value. Data are represented as mean ± SEM, p = 0.5799, 0.5248, and 0.8651 for day 60, day 80, and day 90 two-sample t test, *p < 0.05, **p < 0.01, ***p < 0.001. (C) Representative current responses evoked by a series of voltage steps (from −80 mV to +60 mV, in steps of 10 mV) from organoid (upper) and vOrganoid (lower) cells at day 60, day 80, and day 90. (D) Representative evoked action potentials of cell from organoid and vOrganoid at day 90. The amplitudes of injected currents changed from −60 pA to 280 pA in steps of 20 pA. (E) The percentage of firing cells under current stimulations with different amplitudes. The insertion indicates the evoked action potential of cells from organoids (black) and vOrganoids (red). The scale bars indicate 5 ms and 20 mV. The arrow indicates −20 mV. p = 0.0602, 0.0000025, and 0.00012 for day 60, day 80, and day 90. Data are represented as mean ± SEM, paired-sample t test, *p < 0.05, **p < 0.01, ***p < 0.001, ****p < 0.0001. (F) Resting membrane potentials of recorded cells from organoids and vOrganoids at day 60, day 80, and day 90. Open circles indicate data from individual cells. Filled circles indicate the mean value. Data are represented as mean ± SEM, p = 0.9842, 0.00059, and 0.4102 for day 60, day 80, and day 90, two-sample t test, *p < 0.05, **p < 0.01, ***p < 0.001. (G) Capacitance of recorded cells from organoids and vOrganoids at day 60, day 80, and day 90. Open circles indicate data from ind [file pbio.3000705.s004.tif]

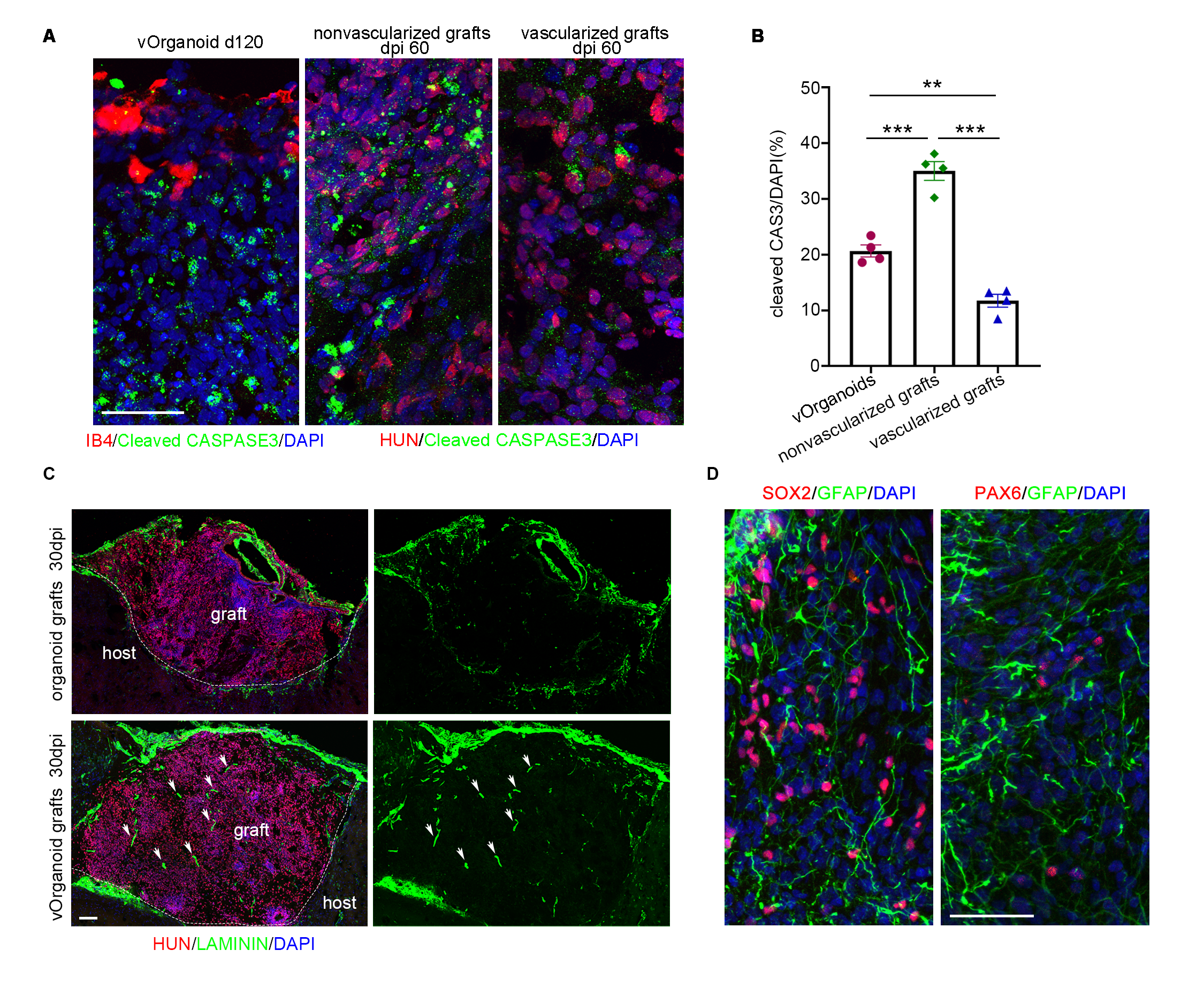

Supplement: S4 Fig — (A) Representative immunofluorescence staining figure for cleaved CASPASE 3, IB4 in cultured vOrganoids at d120 and for cleaved CASPASE 3, HUN in the control nonvascularized organoid grafts and the vOrganoid grafts at 60 dpi. Scale bar, 50 μm. (B) Quantification of the percentages of cleaved CASPASE 3+ cells within all cells (DAPI+) in the cultured vOrganoids at d120, nonvascularized and vOrganoid grafts at 60 dpi, respectively. N = 4, 4, 4 samples for the vOrganoids, the nonvascularized organoid grafts, and vOrganoid grafts from three independent experiments, respectively. Data are represented as mean ± SEM, one-way ANOVA, **p < 0.01, ***p < 0.001. The numerical data underlying this figure can be found in the S4B Fig sheet of S1 Data. (C) Representative immunofluorescence staining figure for LAMININ and IB4 in the cryosections of host brains contained the control organoid and vOrganoid grafts at 30 dpi. The immunostaining of LAMININ is individually displayed in the right panels. The boundary between host and graft is outlined by dashed lines. And the arrows point out the healthy-looking blood vessels in the vOrganoid grafts. Scale bar, 100 μm. (D) Representative immunofluorescence staining figure for GFAP/SOX2 and GFAP/PAX6 in the vOrganoid grafts at 60 dpi. Scale bar, 50 μm. CAS3, CASPASE 3; dpi, days postimplantation; GFAP, glial fibrillary acidic protein; HUN, human nuclear; IB4, isolectin I-B4; PAX6, paired box 6; SOX2, SRY-box transcription factor 2; vOrganoid, vascularized organoid. (TIF) [file pbio.3000705.s005.tif]
