# Supplementary material for: Vascularized human cortical organoids (vOrganoids) model cortical development in vivo
Source: PLoS Biol. 2020 May 13;18(5):e3000705. doi: 10.1371/journal.pbio.3000705 (PMC7250475; doi:10.1371/journal.pbio.3000705)
Supplement: Attachment — Submitted filename: Response to Reviewers.pdf [file pbio.3000705.s012.pdf]

Reviewer remarks:

Reviewer #1: In this study by Shi et al, the authors generate vascularized cerebral organoids (vOrganoids) from human embryonic stem cells mixed with human endothelial cells in vitro. These vOrganoids display the appropriate neural and endothelial cell markers, structural organization, and functionality typically seen in other organoid studies. The authors implant vOrganoids intracerebrally into mouse brain and observe functional connectivity of vOrganoid vessels with the endogenous vasculature indicated by live two-photon imaging of bloodflow and 3-D reconstruction of vessel morphology. Furthermore, the neurons within vOrganoids are able to mature electrophysiologically as shown by patch-clamp electrophysiology and calcium imaging; these data indicate the presence of both excitatory and inhibitory post-synaptic currents as well as gap junction coupling between neurons suggesting the presence of both chemical and electrical synapses in vOrganoids. These data along with the comprehensive single Cell RNA seq data are valuable to other researchers in the organoid field and support the utility of this system. Furthermore, the authors demonstrate that cerebral implantation can support the long-term health and maturation of organoids to better mimic in vivo development. This study is well-designed and rigorous in its evaluation of vOrganoids and their potential for future research. The data provided are in high quality. The study will be of interest to the field of neuroscience and stem cell biology, as well as disease modeling using pluripotent stem cells.

Response: We are grateful for the reviewer's positive views and insightful comments.

Nevertheless, I have the following suggestions may help further improve the study.

1. In the first paragraph on page 7 the authors state "….which may account for the well-developed vascularized organoids with reduced cell death." Can the authors please provide some evidence? Using TUNEL or caspase staining for example.

Response: We completely agree with the reviewer's suggestions. Following the reviewer's suggestion, we performed the CASPASE 3 staining in the organoids cultured with or without HUVECs, respectively. Our results showed that, the cell ratio of CASPASE 3 positive cells in the vOrganoids was significantly decreased from 50.0% to 21.9% as compared to the control non-vascularized organoids (new Fig. S1F and G). The reduction of cell death in the vOrganoids suggest that the HUVECs formed vasculature systems in organoids might improve the cell survival in organoids. The related description was also added in the revised manuscript (Page7-8, Line 151-154).

2. Do the authors have any evidence of synaptic integration between the grafted vOrganoid and mouse brain? Such as colocalization of synaptic markers or via electrophysiology. I recognize that this will be a time-consuming experiment. If sections are available, some immunohistochemistry with high-resolution confocal

imaging analyses could be helpful.

Response: We are grateful for the reviewer's suggestions. Following the reviewer's suggestions, we did the immunofluorescence staining of Synaptobrevin 2 (SYB2) and postsynaptic density protein-95 (PSD95) to review the presynaptic and postsynaptic proteins, respectively, in the 60 dpi grafted mouse brain. The colocalization (arrows pointed out) and close association of SYB2 and PSD95 in the areas of vOrganoid grafts close to the graft-host border indicate robust synaptic connectivity are built between the organoid grafts and the host brain (new Fig. 4C). The related description was also added in the revised manuscript (Page15, Line 304-308).

Minor suggestions.

1. "vOrganoid" needs to be defined in the Abstract section.

Response: We thank reviewer for the suggestions. And we defined the "vOrganoid" in the Abstract section of the revised manuscript.

2. Usages of "GABAA receptor" and "GABAAR"; "NMDA receptor" and "NMDAR"; "AMPA receptor" and "AMPAR" should have some consistency.

Response: We are grateful for the reviewer's suggestions and apologize for not keeping the consistency of using of "GABAA receptor" and "GABAAR"; "NMDA receptor" and "NMDAR"; "AMPA receptor" and "AMPAR" in our papers. We carefully checked the manuscript and kept using "GABAA receptor"; "NMDA receptor"; and "AMPA receptor" in the revised version.

3. Figure 1 panel I is not bolded like the other panel letters.

Response: We corrected it in the revised manuscript (new Fig.1I).

4. What is the relevance of the (#) in the top left of Figure 1 panels I, J, K, L? Statistics should be better defined in figure legend.

Response: We thank the reviewer for pointing these problems out and we apologize for the unclear descriptions. The (#) in the top left of Figure 1 panels I, J, K, L represents the NO. of the continuous sections of organoids. And we added the definition of the (#) in the figure legends in the revised manuscript. As for the statistics, we carefully described the method we used, the number of samples, and p values in the figure legends of the revised manuscript.

5. While infrequent, this study does have some grammatical/syntax errors. I

recommend that the authors stringently go over the manuscript and give it to others with a strong command of English to fix these errors.

Response: We thank the reviewer for pointing this out. We apologize for grammatical errors and have asked native speakers for the grammatical editing.

6. On page 4 the authors state "vRG, oRG, and IPC" acronyms without first defining these for the reader

Response: We thank the reviewer for pointing this out. We added the definitions of "vRG, oRG and IPC" at the first places they appeared in the revised manuscript.

Reviewer #2: Shi et al. present a method to generate vascularized human cortical organoids (vhCOs) by co-culturing human umbilical vein endothelial cells (HUVECs) with human embryonic stem cells (hESCs). They also graft vhCOs into a mouse cortex and demonstrate that the mouse vasculature and human vascular cells join in forming blood vessels. This manuscript complements the existing literature regarding vascularized cerebral organoids and grafting the organoids into the rodent brain, including Pham et al., 2018 and Mansour et al, 2018.

Response: We are grateful for the reviewer's positive views and insightful comments.

While the technique presented in this manuscript may be a viable way to improve on cerebral organoid technologies, the main novelty is the usage of HUVECs instead of stem cell-derived endothelial cells (Pham et al., 2018) and is therefore somewhat limited.

Response: We are grateful for the reviewer's comments. We agree that other than stem cell-derived endothelial cells (Pham et al., 2018), HUVECs is another choice. Pham et al. manuscript illustrated "vascularization of brain organoids with a patient's own iPSC-derived ECs is technically feasible." However, they have not systematically analyzed the developmental features of the culture. In our studies, we characterized the vOrganoids by cell type analysis along the developmental timing in two ways: immunostaining (new Fig.1) and scRNA-seq (new Fig.2). In addition, we also illustrated the receptor maturation, electrophysiological activities and neural network of neurons (new Fig.3). After transplantation, we observed the well-connected blood vessels and robust synaptic integration between the grafted vOrganoid and host mouse brain (new Fig.4).

Other than that, we systematically present the advantages of vascularized organoids over non-vascularized ones (see the detailed response of the next question). Plus, we also used two hES cell lines and two hiPS cell lines to show our methods are reproducible. Third, we compared the scRNA-seq data of cultured vOrganoids and real human developing telecephalon. The results indicate that the growth trajectory of vOrganoids could recapitulate the in vivo development very well.

In summary, based on all of these findings, we think we have provided a more reliable method to establish the vascularized cerebral organoid culture technologies.

In addition, some of the conclusions in the manuscript are somewhat tenous. Firstly, the claim that the vhCOs present an advantage over non-vascularized ones was not substantiated. Secondly,the claim that this method is highly reproducible was not supported by the data. The study is also somewhat lacking in rigor and statistical

analysis.

Response: We are grateful for the reviewer's comments. In the revised manuscript, we added experimental data and also rewrote the text to make the points that reviewer mentioned clear.

(1) Firstly, we systematically compared the control organoids and vOrganoids (co-culture with HUVECs) and found vOrganoids presented several advantages over the non-vascularized ones: (a) improved cell survival in vOrganoids during culture (new Fig S1F-G) and larger size (new Fig.S1H); (b) increased thickness of the neural progenitor cells zone (new Fig S1I-K) and enhanced neurogenesis. The enhanced neurogenesis was illustrated by scRNA-seq and immunostaining, respectively, at day 65 (new Fig. 2I-J); (c) earlier maturation of the active electrophysiological activities (new Fig 3A-B and Fig.S3A-B); (d) improved cell survival in the transplanted grafts (S4A-B).

(2) Secondly, we used two human ES cell lines (H9 and H3) and two human iPS cell lines to test the reproducibility of the method. All of these cell lines could form vOrganoids with high successful rate (Fig S1D-E). We added related description in the revised version. (page 7, line 145-151).

(3) All the immunostaining images are representative data. We have added the description of how we collect the samples, n numbers and statistical analysis in the revised manuscript, especially in figure legends and methods (For example, Page12 Line 249-251; Page 29, Line 669-671; Page 31, Line 719-727).

The major concerns are as follows:

1) One of the main arguments of the manuscript is that the vascularized organoids provide an advantage over non-vascularized ones. The authors found that vascularized organoids are slightly larger in Fig S1E, but this is not inherently an advantage. Moreover, they claimed that there is a relatively small proportion of Caspase-3 positive apoptotic cells in grafted vhCOs (Fig S4A), but there is no control group for comparison. More importantly the authors did not present data on Caspase-3 or TUNEL staining of ungrafted vhCOs grown in parallel to non-vascularized ones to demonstrate a decrease in cell death that would support their claim of reduced cell death. Also, no markers for hypoxia were included to support the claim of better oxygenation.

Response: We thank the reviewer for the comments. Following the reviewer's suggestion, we carried out more experiments and made the following revision.

(1) We performed the CASPASE 3 staining in the organoids cultured with or without HUVECs before transplantation. Our results showed that, the ratio of CASPASE 3 positive cells in the vascularized organoids with HUVECs was significantly decreased from 50.0% to 21.9%, compared to the non-vascularized organoids without HUVECs

(new Fig. S1F-G). The related description was also added in the revised manuscript (Page7-8, Line 151-154).

(2) Hypoxia inducible factor 1 subunit alpha (HIF1α) is one reported hypoxia marker(Hutchison et al., 2004). We added the HIF1α staining in the organoids cultured with or without HUVECs. The results showed that the HIF1α positive cells in the organoids with HUVECs mainly presented in the center part. However, in the organoids without HUVECs, the HIF1α positive signals were densely distributed in the whole sections (new Fig.S1F-G). The distribution was in consistence with that of CASPASE 3 staining. Therefore, our results indicate that the vascular systems in the vOrganoids might support better oxygenation as compared to the non-vascularized organoids. The related description was also added in the revised manuscript (Page 8, Line 154-159).

2) The authors also claimed that based on single cell RNAseq data, the vhCOs "exhibited microenvironments to promote neurogenesis and neuronal maturation that resembled in vivo processes." While the scRNAseq showed the presence of neurogenesis and neuronal maturation, it cannot demonstrate the presence of microenvironments or stem cell niches. The authors stated that the presence of microglia and choroid plexus cells in vhCOs provides a more complex microenvironment that contributes to neurogenesis, but this feature is not unique to the vascularized organoids, and they present no data showing that this contributes to neurogenesis.

Response: We added two biological replicates in scRNA-seq data and carefully looked at the microglia and choroid plexus cells. We agree with reviewer's comments that these cells were not just from vOrganoids, so we removed this statement in the revised manuscript.

3) The authors suggest that the vhCOs had higher maturation scores in Fig. 2H-I, but this is not evident from Fig. 2I. Rather, the cells from the vhCO seem to be scattered around and may be more highly represented among the lower maturation scores. No statistical analysis was presented for these data. The authors also referenced Fig. S2E and claimed that the vhCOs are more mature, but this figure does not seem to compare vascular and non-vascularized hCOs.

Response: We thank the reviewer for pointing this out. In the revised manuscript, we removed the comparison of maturation scores between the vascularized organoids and the non-vascularized ones. Instead, we have added the comparisons of neurogenesis and electrophysiological activities between neurons of vascularized organoids and the non-vascularized ones (new Fig. 2I-J and Fig. 3A-B, S3A-B).The scRNA-seq and immunostaining analysis demonstrated that the ratio of NEUROD2$^+$excitatory neurons were significantly higher in vOrganoids, indicating more neurogenesis occurred in the vOrganoids at early time (new Fig. 2I-J). Besides, the direct batch to batch comparisons

of the inward currents and spontaneous action potential, indicate that the vOrganoid neurons were electrophysiologically more mature than the non-vascularized ones. The related description was also added in the revised manuscript (Page11, Line 225-228, Page 12-13, Line 244-253).

4) The authors state that the vhCOs are electrophysiologically more mature because they were able to obtain Na+ currents and spontaneous action potentials at 90-100 days in vitro, earlier than in their prior work without endothelial cells. There was no parallel control with non-vascularized organoids, and therefore one cannot conclude that the presence of vasculature speeds functional maturation of neurons without direct batch-to-batch comparison.

Response: We are grateful for the review's comments. Following the reviewer's suggestion, we performed new experiments to do the direct batch-to-batch comparison between the vascularized organoids and the non-vascularized organoids at day 60, 80 and 90. The results showed that the vOrganoids exhibited significantly larger amplitudes of outward currents from day 80, while the sodium currents were almost at the same level (new Fig. 3A,B, S3A). Since outward currents have increased significantly during the maturation process of cortical neurons (Guan et al., 2011), our observation suggested the neurons of vOrganoids might be more mature than that of non-vascularized organoids. Consistently, more neurons showed spontaneous action potentials at day80 (day 80 is the earliest timepoint we could detect spontaneous action potential) in the vOrganoids (8/56 cells, 14.3%) than in the non-vascularized ones (4/52 cells, 7.7%) (Figure S3B), indicating that the vascular system in the vOrganoids may accelerate the progression of the functional development of individual neurons in vitro.

5) The claim that this method is highly reproducible was not supported by the data. The authors note that they used 2 different human embryonic stem cell lines, but it is unclear which experiments were done with which cell line. Fig S1D shows both cell lines, but are all the other experiments from a single line? In addition, throughout the manuscript, details on how many times a certain experiment or differentiation were performed were not included. For example, what proportion of the vhCOs successfully exhibited vascular structures? To demonstrate reproducibility, multiple differentiations should be performed from multiple cell lines with consistent results.

Response: We are grateful for the reviewer's comments and apologize for not describing clearly. We actually did tests on many cell lines. Other than H3 and H9 (new Fig. S1D), we added the results of two hiPS cell lines (new Fig. S1E). We repeated H9 line for more than 30 batches from at least 3 different people. For other cell lines, we

repeated more than 3 batches. In our hands, >95% proportion of the vOrganoids successfully exhibited vascular structures in the H3 and two iPS cell lines. And we have 100% success rate in H9 cell line. The vascular and cortical structure analyses have been done in organoids from all the cell lines. The scRNA-seq, electrophysiology experiments and transplantation experiments were used H9 cell lines. We include this information in the revised manuscript in the method part of "3D vOrganoids culture and differentiation procedure". (Page 40, Line 950-953).

6) For scRNAseq, how many organoids were analyzed? The experiment was not set up to examine variability between organoids or between batches of organoids as other published studies have done to look at reproducibility (e.g Velasco et al, Nature, 2019 and Yoon et al., Nature Methods, 2019).

Response: We are grateful for the reviewer's comments. We added two more batches of scRNA-seq experiments into our original study (three batches in total in the revised manuscript). As for the number of organoids analyzed in the scRNA-seq data, at least ten organoids were involved for each scRNA-seq experiment. Since we had twelve independent scRNA-seq experiments (new Fig.S2A, organoids d65_01/02/03; vOrganoids d65_01/02/03; organoids d100_01/02/03; vOrganoids d100_01/02/03) in this studies, therefore we have at least 120 organoids involved in the scRNA-seq studies.

7) For electrophysiology, what was the success rate for patching cells? Of the cells that were successfully patched, what proportion had spontaneous activity and sodium currents?

Response: We thank the reviewer for pointing this out. We performed whole-cell recording on 330 cells from different developmental stages. Sodium currents have been identified from 271 cells (82.1%, 271/330). Spontaneous activities (either spontaneous spikes or spontaneous EPSCs/IPSCs) have been observed from 106 cells (32.1%, 106/330). The information was updated in the figure legends.

8) There are a number of concerns with the in vivo grafting experiments. First, how many times were the grafting experiments done? In the few times that the authors state "n=…" it is unclear if the "n" refers to independent experiments or rather 3 samples from 1 experiment. For example, in Fig. 1M, n=3 samples may mean 3 organoids from a single differentiation, rather than 3 independent differentiations. For Fig 3E and 3G, n=6 was for 6 cells, which may be all from the same organoid. Second, the grafted organoid shown in Fig. 4 appears to be outside the parenchyma of the mouse brain, which it deforms from mass effect. Third, the authors claim laminar organization with CTIP2 being deeper and SATB2 being more superficial. This is not readily evident in Fig. 4B, and moreover, there appear to be

DAPI+/CTIP2-/SATB2- rosette structures superficial to the demarcated SATB2 cells. If this recapitulated normal laminar organization, the rosette should be deep to the neurons.

Response: We are grateful for the reviewer's comments. First, we apologize for the unclear descriptions. Actually, n means the number of independent experiments from different biological repeats. We have clearly described the meanings of n number in the figure legends of the revised manuscript.

Second, we think the protrusion of the grafted organoid outside the parenchyma of the mouse brain may result from the continuous growth of grafted organoids in the host brain.

Third, we agree with the reviewer's comments that there are the DAPI+/CTIP2-/SATB2- rosette structures located superficial to the demarcated SATB2 positive cells, which is similar to the rosette structures in the grafted organoids from other studies (Mansour et al.,2018). We apologized for not updating progress to our manuscript since we just transferred our manuscript from Cell Stem Cell to Plos Biology. We restated this observation in the revised manuscript and updated our reference (Page16, Line 320-325).

9) In the grafting experiment shown in Fig 4E, the authors state that there are GFAP+ cells in the grafts that appear to be derived from the human ES cells. They should discuss the discrepancy on why they find GFAP+ human cells in the grafts, but not in vitro when examined by scRNAseq. Furthermore, the GFAP staining in Fig 4E appears linearly aligned, which would be unusual for parenchymal astrocytes or astrogliosis, and instead may reflect radial glia - other markers for radial glia should be used to exclude this possibility. Also, the authors state that the myelinated fibers from the mouse are intruding into the graft. How can this be distinguished from the graft intruding into the mouse brain?

Response: We are grateful for the reviewer's comments. We added 2 more batches of sequencing data of each group in our revised manuscript. With more cells, clustered astrocytes were detected in the updated scRNA-seq data (new Fig.2A), which is in consistence with the results of immunostaining (new Fig. 2G) and intracerebral implantation experiments (new Fig. 4F). To exclude the possibility of the GFAP positive cells in the grafted organoids might be radial glia, we performed the immunofluorescence staining of PAX6 and GFAP staining. It showed that very few GFAP/SOX2 and GFAP/PAX6 co-labelling cells could be detected (new Fig.S4D), indicating that the majority of $GFAP^+$ cells in the grafts were astrocytes.

After transplantation, we observed abundant MBP positive myelinated fibers in the host brain, and sparse myelinated fibers in the grafted organoids that distributed along the graft-host border (new Fig.4G). Given that no oligodendrocytes was detected in our scRNA-seq data or staining of organoid grafts, we think that it is more likely the myelinated fibers from the mouse are intruding into the graft but not the graft-derived myelinated fibers intruding into the mouse brain.

Other concerns:

10) The authors should discuss their choice of using HUVECs over other endothelial cells, including stem cell-derived endothelial cells, and in particular brain microvascular endothelial cells, which would seem to be a more appropriate choice.

Response: Following the reviewer's suggestions, we added descriptions of the characterization of HUVECs in the revised manuscript (Page 6, line 117-121). HUVECs (human umbilical vein endothelial cells), derived from the endothelium of veins from the umbilical cord, were first isolated and cultured in vitro in the 1970s by Jaffe and others (Jaffe et al., 1973). They are widely used to explore the function and pathology of endothelial cells (e.g., angiogenesis) (Chen et al., 2009; Nakatsu et al., 2003). Via co-culturing with other cell types, HUVECs were extensively used to characterize the angiogenesis during tumorigenesis (Choudhuri et al., 1997; Okuda et al., 2003). Besides, HUVECs can be easily made to proliferate in a laboratory setting for use.

Human brain microvascular endothelial cells (HBMECs) play important roles in the development of blood-brain barrier (BBB) (Kuo and Lu, 2011). We examined that whether the co-culture induces the HUVEC cells to develop towards a more brain like endothelial cell fate, such as HBMECs. P-gp is a well-characterized ATP-binding cassette (ABC)-transporter that expressed at the luminal membrane of the brain capillary endothelial cells (Daood et al., 2008). Hence, we performed the immunostaining of P-gp (P-glycoprotein) in the vascularized organoids at day 83. Our results showed that P-gp was co-localized with IB4 to a great degree (new Fig.S1L), suggesting that the co-culture in vOrganoids might change the gene expression of HUVECs to more brain like endothelial cells.

In summary, given the following four points: (1) HUVECs could be well-connected into the elaborate mesh or tube-like vascular systems in the vOrganoids, which greatly improve the cell survival rate in vOrganoids (new Fig.1C, S1B, S1F-G). (2) The vascular systems built by HUVECs were highly repeatable in different hESC and hiPSC

lines (new Fig. S1D-E). (3) HUVECs could be induced to develop towards the HBMEC-like cells by co-culture (new Fig.S1L). (4) HUVECs is easy to be maintained in a laboratory setting for use. We think the choice of HUVECs to generate the vOrganoids is reasonable and feasible.

11) In the introduction, the authors should consider citing cortical and cerebral organoid papers from the Sasai, Lancaster/Knoblich, Pasca, Song Labs (instead of or in addition to midbrain and hippocampal organoid papers). Also, the Mansour et al. paper from the Gage lab should be cite earlier in the introduction. The manuscript authors state that there has been no sophisticated vascular structure observed in brain-like organoids, but the Mansour et al. paper showed this.

Response: We thank the reviewer for pointing this out. In the revised manuscript, we cited the cortical and cerebral organoid papers from the Sasai, Lancaster/Knoblich, Pasca and Song labs in addition to the papers related to midbrain and hippocampal organoid. And we also cited the papers of Mansour (Mansour et al., 2018) earlier in the introduction in the revised manuscript (Page 5, Line 94-96).
We apologize for the description of "there has been no sophisticated vascular structure observed in brain-like organoids". And we removed this sentence in our revised manuscript.

12) In Fig. 1C, the tube-like structures are not appreciated well, and in Fig. 1D, the TBR2+ layer appears thin.

Response: We appreciate the reviewer's comments. At day 45, the large proportion of cells are neural stem cells ($SOX2^+$ $TBR2^-$cells) but not $TBR2^+$ cells, so TBR2+ layer appears thin in the new Fig.1D. As showing in the new Fig.1H (left panel), there are more $TBR2^+$ cells in the vOrganoids of day65, suggesting $TBR2^+$ layer might become gradually thick in the early development.

Also, it is unclear why there should be any cells triple-labeled with Tbr2/Sox2/IB4, as IB4 labels the vascular structures, not the intermediate progenitor cells.

Response: We thank the reviewer for pointing this out. In fact, there are no cells triple-labeled with TBR2/SOX2/IB4, the suspected triple-label in Fig.1D resulted from the overlap of the cells. And we apologize for the previously improper description in the figure legends. In the revised manuscript, we removed the arrow and arrowheads in the new Fig.1D as well as the related description in the figure legends.

13) In Figure 1E, Sox2 and p-VIM will label mitotic ventricular radial glia, and this

is not specific to outer radial glia. The authors should use another marker such as HOPX for outer radial glia. Also, this staining was done at d65, an age at which on scRNA seq there were no HOPX+ outer radial glia.

Response: We are grateful for the reviewer's comments. Following the reviewer's suggestions, we performed the immunofluorescent staining of HOPX/SOX2/IB4 at day 65. And the staining results verified the presence of HOPX positive outer radial glia (oRG) in the vascularized organoids at day 65 (new Fig 1E). What's worth noting is that, the $HOPX^+$ oRGs are also detected in our updated scRNA-seq data of day 65 (new Fig. 2A). Thus, the results of scRNA-seq and the immunofluorescent staining both corroborated the emergence of oRG in the cultured organoids.

14) Fig 1K-M: Reelin and Cux1 immunolabeling would be helpful to show superficial cortical layer markers.

Response: We are grateful for the reviewer's suggestion. And following the suggestion, we performed the immunostaining of RELN in our studies. The results of which showed that the early-born $RELN^+$ cells were located on the superficial layer (new Fig S1O). Besides, we used two upper-layer markers, STAB2 and BRN2 (new Fig 1J-M, Fig.S1N), and two deep-layer markers, CTIP2 and TBR1 (new Fig. 1H, K-M, S1O).

15) The lack of astrocytes on scRNA is puzzling. This should be confirmed with GFAP staining of the organoids.

Response: In the revised manuscript, we added 2 batches of scRNA-seq data in each group so we have 57180 cells from 12 independent samples. In the updated scRNA-seq data, clustered astrocytes were detected (new Fig.2A). Besides, the immunofluorescent staining of GFAP in organoids at d65 also confirmed the emergence of astrocytes in vOrganoids (new Fig.2G). The reason why we did not find clustered astrocytes could be the cell number limitation.

16) In Fig. 2B, it appears that there are many more cells in the vOrganoids at d100 and d65 compared to the unvascularized organoids. This may be because the plot has opaque dots for the vOrganoids that are overlayed on top of the dots for the unvascularlized organoids. tSNE plots showing the vOrganoids and unvascularized organoids separately would help visualized these data. This also applies to Fig 2I.

Response: We are grateful for the reviewer's comments. Following the referee's suggestions, we exhibited the data of four types of organoids separately in our revised manuscript (new Fig.2B). According to the response to your previous question, we removed the contents of comparisons of maturation score between vascularized and non-vascularized organoids in the revised manuscript.

In addition, for the gene ontology analysis in Fig. 2F, are there several marker genes that fall under each category? If so, brackets should be used to show which rows of the heatmap correspond to the GO class. If not, having just 1 enriched synaptic gene (SYT1) does not make a compelling argument that vOrganoids are enriched in synaptic markers.

Response: We thank the referee for pointing this out. There are several DEGs exist in the enriched GO terms. We added a spreadsheet in Table S1 to list the enriched GO terms and the related DEGs.

17) Fig 2D: The choice of comparing the organoid scRNAseq data to a dataset containing 2300 cells from human fetal prefrontal cortex from gestational ages (GW8-26) should be explained. The samples are restricted to a specific brain region. The dataset from Nowakowski et al., 2017 may be a better comparison.

Response: We are grateful for the reviewer's comments. Given the organoids generated in our studies are meant to recapitulate the paradigms of human cortical development *in vitro,*

we tried to compare the organoid scRNA-seq data to the dataset focused on human cortical development. Therefore, we chose the dataset of human fetal prefrontal cortex to do the comparison from Zhong et al., 2018. However, we agree with the reviewer's suggestions that the dataset from Nowakowski et al., 2017 could be a better choice because it contains additional visual cortical areas across stages of peak neurogenesis. Therefore, in our revised manuscript, we did the comparison between the scRNA-seq data of organoids and the developing telencephalon from Nowakowski paper. The results showed high similarity between the cell types of cerebral organoids and human developing cerebral cortex, indicating the cultured cerebral organoids could recapitulate the paradigms of human brain development. The details were demonstrated in the revised manuscript (Page 10-11, Line 203-213).

18) Fig 2D and S2D: The interneuron populations of the organoids/vOrganoids do not overlap well with the human prefrontal cortex samples on the tSNE plot. The authors should comment on this and determine what genes may be differentially expressed between these two groups.

Response: We thank the reviewer for pointing this out. We added 2 more batches of organoid scRNA-seq data in the revised version. And in the updated scRNA seq data of organoids, we detected a group of interneurons highly expressed the classical interneuron gene markers, such as GAD1, DLX1 (new Fig.2A and S2C). And by comparing this interneuron cell group to the human cortical interneurons, it showed that

the two groups of interneurons could match to each other very well (new Fig.2C-E). The previous non-overlap results of two interneuron populations in the original version may be due to the low cell number, which is revised in this version.

Also, it is not clear if the interneurons derive from ganglionic eminence-like progenitors – markers for these (e.g., NKX2.1, DLX1/2, LHX6) should be examined by immunostaining and scRNAseq.

Response: We are grateful for the reviewer's insightful comments. And following the reviewer's suggestions, we firstly checked the expression of markers of progenitors of ganglionic eminence in scRNA-seq analysis, such as NKX2.1, DLX1/2, LHX6. It showed that DLX1 were highly expressed in the cell group of interneurons (new Fig.S2C). Besides, we also detected sparse expression of NKX2-1 and LHX6 (the downstream gene of NKX2.1) in the scRNA-seq data (new Fig.S2C). Then we performed the immunostaining of NKX2.1 in the organoid sections, and in consistence with the results of scRNA sequencing, a few of NKX2.1^{+} cells were detected (new Fig.2H).

19) Fig 2H-I: The maturation trajectory does not appear to be tightly correlated compared to the cited study (Mayer et al, 2018).

Response: We are grateful for the reviewer's comments. The maturation trajectory in the studies of Mayer et al.,2018 was used for cells from ganglionic eminence. In our data, it applied for cortical analysis, which may be not very suitable. Following reviewer's suggestion, we removed the part of maturation score analysis in our revised manuscript.

20) For sodium/membrane current recordings (Fig 3A and S3D), additional steps to other voltages should be included (not just +20 and -20 mV).

Response: Following the reviewer's suggestions, we added more traces at different voltages in the new Fig.3A and new Fig.S3A, F.

21) The authors stated in the discussion "Pyramidal excitatory neurons and interneurons in the vOrganoids were aligned in layers that were similar to those of human neocortical lamination." This was not demonstrated by the data.

Response: Following the reviewer's suggestions, we added an immunostaining of human developing cortex at gestation week 23 (GW23) to show the lamination, SATB2 for the upper layer neurons and FOXP2 for the deep layer neurons (new Fig.S1M).

22) Fig S1G: It is unclear what the white and black bars represent, and they are switched in position between the top and bottom panels.

Response: We thank the reviewer for pointing this out. In the revised manuscript, we have repositioned and explained what the white and black bars represent (new Fig.S1J-K).

23) In the asbstract, the "C" in sEPSCs and sIPSCs stands for current, not potential.

Response: We have corrected the annotation of sEPSC and sIPSC to "spontaneous excitatory postsynaptic current" and "spontaneous inhibitory postsynaptic current" in our revised manuscript.

Reviewer #3: Shi et al develop a cerebral organoid differentiation protocol where they achieve generation of the vasculature (vOrganoids) by co-culturing HUVEC cells with human ESCs differentiated into cerebral organoids.

Overall, the manuscript is poorly prepared. Many descriptions lack proper quantifications, and many quantifications lack proper statistical reporting. There are numerous grammatical errors throughout the manuscript, and the logic of the experiments is difficult to follow.There are several potentially interesting findings. For example, it is interesting that the vasculature localizes to the SVZ and observing spontaneous action potentials by day 90 suggests the vOrganoids mature well. Similarly, observing microglia in a forebrain protocol is unexpected and potentially useful.However, my biggest concern that the authors have not really convincingly showed that their method of vOrganoids cultures have any major benefits over existing protocols.

Response: We are grateful for the referee's sharp comments.

Major concerns,
1) Despite a long introduction, the authors do a very poor job reviewing the state of the field of vascularization in organoids. For example, there is only a brief description of Mansour et al., 2018 in the discussion, and Pham et al., 2018 is only briefly mentioned in the introduction. Moreover Daviaud et al., 2018 is not really described.

Response: We are grateful for the reviewer's comments. In the revised manuscript, we cited and discussed about the studies of Mansour (Mansour et al., 2018) , Pham (Pham et al., 2018), and Daviaud (Daviaud et al., 2018) in details (Page4-5, Line 91-99).

2) The main point of the article is the role of endothelial cells, yet there is very little characterization of these cells.

Response: We are grateful for the reviewer's insightful comments. And following the reviewer's suggestions, we added the description of the characterization of HUVECs in our revised manuscript (Page 6, Line 117-121). HUVECs (human umbilical vein endothelial cells), derived from the endothelium of veins from the umbilical cord, were first isolated and cultured in vitro in the 1970s by Jaffe and others. They are widely used to explore the function and pathology of endothelial cells (e.g., angiogenesis). Via co-culturing with other cell types, HUVECs were extensively used to characterize the angiogenesis during tumorigenesis (Chen et al., 2009; Choudhuri et al., 1997; Okuda et al., 2003). Besides, HUVECs can be easily made to proliferate in a laboratory setting.
These cells seem to have not been captured by the scRNA-seq, but could be characterized by other methods.

Response: We are grateful for the reviewer's comments. The tight junctions existed between endothelial cells play pivotal roles in tissue integrity, barrier function and cell–cell communication, respectively(Huber et al., 2001). However, the mild dissociation method used to prepare the single-cell suspension of organoids may not validly disassemble the tight junctions. This could be a reason for why endothelial cells were not captured in the scRNA-seq data. But the endothelial cells derived from HUVECs could be widely detected by immunostaining, which confirmed the actual existence of endothelial cells in the vascularized organoids. In addition, GO terms of DEGs between vOrganoids and non-vascularized organoids from scRNA-seq analysis are related to blood vessel morphogenesis and cell response to oxygen level (new Fig.2K), suggesting there might be some endothelial cells but not clustered due to limited cell number caught by scRNA-seq. These GO terms also indicate that the vasculature development may also change the gene expression in other types of cells to respond to oxygen levels.

In particular, HUVEC cells are likely very different from endothelial cells in the brain, but perhaps co-culture induces the HUVEC cells to a more brain like endothelial cell fate. This should be examined, and the authors should clearly acknowledge the differences between these cells and endothelial cells of the brain.

Response: We are grateful for the reviewer's insightful comments. We agree with the reviewer's viewpoint that HUVECs were different from the human brain microvascular endothelial cells (HBMECs), as HBMECs play important roles in the development of blood-brain barrier (BBB). Following the reviewer's suggestions, we examined that whether the co-culture could induce the HUVEC cells to a more brain like endothelial cell fate. P-gp (P-glycoprotein) is a well-characterized ATP-binding cassette (ABC)-transporter that expressed at the luminal membrane of the brain capillary endothelial cells(Daood et al., 2008). We performed the immunostaining of P-gp in the vascularized organoids at day 83. Our results showed that P-gp was co-localized with IB4 to a great degree (new Fig.S1L). These results indicate that the co-culture might induce the HUVECs develop towards a more brain like endothelial cell fate.

3) Throughout the manuscript, the authors provide insufficient information about many of the methods used for quantifying the phenotypes. Many descriptions appear to reflect some kind of quantification but no quantification or statistics is shown. For example, quantification of organoid growth presented in Figure S1 E – it is completely unclear from the figure description how many individuals were used, how many organoids, how were they selected, what statistical method was used for comparison, was there multiple hypothesis correction, what was the data distribution. This is just one of many examples where reporting on data quantification is unacceptable and makes it impossible for me to evaluate the statistical rigor in this

manuscript.

Response: We thank the reviewer for pointing this out, and apologize for not providing the sufficient information about the methods and statistics. And following the reviewer's suggestions, we carefully checked all the quantifications and statistics in our studies, and clearly described what statistical method was used; how many individuals were used, how many organoids were used in the figure legends of the revised manuscript.

4) In addition, I was expecting extensive characterization of the strengths and weakness of vOrganoids vs. conventional organoids, but the comparisons are limited. In particular, how much is cell death reduced in organoids, and do the vOrganoids survive transplant better than conventional organoids?

Response: We are grateful for the reviewer's comments. We agree with the reviewer, and in the revised manuscript, we systemically compared the non-vascularized organoids and vOrganoids and found vOrganoids presented several advantages over non-vascularized ones as follows: (a) improved cell survival in vOrganoids during culture (new Fig S1F-G) and formed larger size (new Fig.S1H); (b) increased thickness of the neural progenitor cells (new Fig S1I) and enhanced neurogenesis. The enhanced neurogenesis was illustrated by scRNA-seq and immunostaining, respectively at day 65 (new Fig.2I-J); (c) earlier maturation of the active electrophysiological activities (new Fig 3A-B and Fig.S3A-B); (d) improved cell survival in the transplanted grafts (Fig S4A-B).

In regarding to cell death analysis, we performed the immunostaining of CASPASE3 in the vascularized and non-vascularized organoids. It showed that the number of CASPASE3^{+} apoptotic cells in the vascularized organoids was significantly decreased from 50.0 % to 21.9%, as compared to the non-vascularized organoids (new Fig. S1F-G).

Besides, we also performed the immunostaining of CASPASE 3 in the grafted vascularized and non-vascularized organoids after transplantation. It showed that as compared to the non-vascularized organoid grafts, the cell death in the vascularized grafts were decreased, which may indicate that the vascularized organoid grafts could survive better than the non-vascularized organoids after transplantation (new Fig.S4A-B). Besides, we also compared the ratio of the CASPASE3^{+} cells in the cultured vOrganoids at day 120 (the number of days corresponding to the 60dpi of organoids transplanted at day 60) to the vascularized and non-vascularized grafts. It showed that the CASPASE3^{+} cells in the cultured vOrganoids of day 120 were significantly decreased as compared to the control non-vascularized grafts, and were increased as compared to the vOrganoid grafts (new Fig.S4A-B). These results suggest that the

vascular systems in the vOrganoids could improve the cell survival in vitro. And the vOrganoid grafts in the host brain would furtherly improve the cell survival rate.

For example, in In Figure S4 the authors show a Caspase3 stain but nothing to compare it too. Are there many Cas3 positive cells in organoids to begin with? Could they provide some quantification in organoids that were and were not transplanted into mouse?

Response: We are grateful for the reviewer's comments. And following the referee's suggestions, we performed the immunostaining of CASPASE3 in the organoids that were and were not transplanted into mouse and quantified the number of CASPASE3$^+$ cells (new Fig.S4A-B). It showed that the CASPASE 3$^+$cells in the cultured vOrganoids of day 120 were significantly decreased as compared to the control non-vascularized grafts, and were increased as compared to the vOrganoid grafts (new Fig.S4A-B).

Similarly, is there more blood flow into vOrganoids than conventional organoids after transplant? Could the size increase of vOrganoids be driven by expansion of HUVECs themselves? There are some differentially expressed genes, but these should be validated by immuno and quantification across normal and vOrganoids.

Response: We thank reviewer for the comments. Since we could not precisely measure the volume of blood flow via analyzing the videos captured by two-photon imaging, we could not provide the direct quantification data to demonstrate whether there is more blood flow into the vascularized organoids than the non-vascularized ones. Instead, we performed immunostaining of IB4 in the vascularized and non-vascularized organoid grafts, which showed that there are more vascular-like tubes in the center of the vOrganoid grafts than the non-vascularized grafts at 30 days after transplantation (new Fig.S4C).

From scRNA-seq data analysis and immunostaining, we did not see cell type differences between vascularized and non-vascularized organoids (new Fig 2A-B). However, lots of DEGs were detected between the non-vascularized and vOrganoids, such as NEUROD2, one of the DEGs of the vOrganoids. scRNA-seq analysis and immunostaining of NEUROD2 at day 65 both verified that more NEUROD2 were expressed in the vOrganoids (new Fig. 2I-J), suggesting the neurogenesis in vOrganoids was enhanced as compared to the non-vascularized ones. Other than that, fewer cell death could be another reason for the size increase in vOrganoids.

5) At the end of the first results section the authors claim that "higher degree of

vasculature assures sufficient oxygen and nutrient support for cell proliferation and differentiation." However, it is unclear to me how this could possibly be achieved simply after co-culturing HUVECs. If the authors believe that this is the case they should address this experimentally. For example, if the hollow tubing of vasculature supplies more oxygen, this is testable.

Response: We are grateful for the reviewer's comments. Hypoxia inducible factor 1 subunit alpha (HIF1α) is one reported hypoxia marker. We added the HIF1α staining in the organoids cultured with or without HUVECs. The results showed that the HIF1α$^+$ cells in the vOrganoids were mainly presented in the center part. However, in the non-vascularized organoids, the HIF1α positive signals were densely distributed in the whole sections (new Fig.S1F-G). Even with this evidence, we think this could not directly verify that "higher degree of vasculature assures sufficient oxygen and nutrient support for cell proliferation and differentiation". So we restated this conclusion in the revised manuscript (Page 8, Line 154-163).

6) Description of electrophysiological properties appears to be performed well, but it is unclear what novel observations are made. This component would be strengthened by experiments showing differences between the vOrganoids and standard organoids.

Response: We are grateful for the reviewer's insightful comments. And following the reviewer's suggestion, we did batch-to-batch comparisons between the vascularized and non-vascularized organoids by performing the whole-cell recording experiments at day 60, 80 and 90. It showed that vOrganoids cells exhibited significantly larger amplitudes of outward currents than the control non-vascularized organoids cells from day 80, while no significant difference was detected in inward sodium currents (new Fig.3A-B and S3A). Since outward currents have increased significantly during the maturation process of cortical neurons (Guan et al., 2011), our observation suggested the neurons of vOrganoids might be more mature than that of the non-vascularized organoids. Consistently, more neurons showed spontaneous action potentials at day80 (day 80 is the earliest timepoint we could detect spontaneous action potential) in the vOrganoids (8/56 cells, 14.3%) than in the non-vascularized ones (4/52 cells, 7.7%) (Figure S3B), indicating that the vascular system in the vOrganoids may accelerate the progression of the functional development of individual neurons in vitro.

7) Most of the data showing human cerebral organoid cells after transplantation are very premature and mostly show the presence of different cell types that are normally found in an organoid after long enough culture. Integration into the mouse tissue is not novel and has been reported before.

Response: We are grateful for the reviewer's comments. Cell death is a big problem

for transplantation (Fischer et al., 2000; Wekerle et al., 2001). In our studies, we found there are more vascular-like tubes in the center of the vOrganoid grafts than the non-vascularized grafts at 30 days after transplantation (new Fig.S4C). Plus, fewer CASASE3^{+} cells in vascularized grafts (new Fig.S4A-B), indicating the vascular systems in the vOrganoids may help grafts survive.

Minor concerns

- Saying that organoids are laminated is quite a stretch. The reality is that there is a clear separation of progenitors vs neurons but within the neuronal layer having “cortical ” -like layers has not been perfected. For the point of this article lamination is not relevant so I would recommend removing references to it.

Response: Following the reviewer’s suggestions, we toned down the claims of lamination in our studies. Besides, we removed the related references.

Moreover, their definition of upper layer neurons is SATB2+ cells, but this marker labels an identity (callosal neurons of all layers) not a layer. Similarly, CTIP2 labels subcerebral projection neurons.

Response: We are grateful for the reviewer’s comments. There are lots of published studies used SATB2 and CTIP2 to label the upper and lower neuronal layers, such as the work of Qian et al., 2016 (Qian et al., 2016). Other than these two markers, we also added staining of BRN2 for upper layer and TBR1 for deep layer neurons (new Fig.1L, S1O). In addition, Layer1 neurons was reviewed by RELN staining (new Fig.S1O).

- The appearance of microglia was reported in one other study (Ornel et al., 2018), but is a bit mysterious. Does this also occur in conventional organoids by this group or is it related to the HUVEC cells? Can the authors explain this result further? How are these distinguished from macrophages?

Response: The microglia could be detected in the vascularized organoids as well as the non-vascularized organoids by scRNA-seq, indicating that the microglia emergence are not due to adding HUVEC cells. Since we used hESC and hiPSC for the culture, all of which have pluripotent properties, so the micrglia could come from them. However, we could not provide any direct evidence to explain the potential reasons for the emergence of microglia in the cultured organoids. We did not distinguish the microglia from macrophages in our studies.

Also, staining for AIF1 in figure 2 seems to be present almost everywhere in the field of view. It is unclear whether the staining was specific to AIF1.

Response: We thank the reviewer for pointing this out. We think the AIF1staining is specific because they showed cellular shape other than random signals (new Fig.2F). Besides, the AIF1 positive signals could be captured in organoids from different independent experiments. We replaced the picture of AIF immunostaining to the one with cleaner background in the revised manuscript (new Fig.2F).

- "The transplantation of cerebral organoids that are derived from hESCs or iPSCs into injured areas may be a promising therapy for improving neurologic deficits that are caused by trauma or neural degeneration" . I do not think there is a need for this sentence in the manuscript. What evidence do they have that transplanting an heterogeneous tissue (vs for instance transplanting pure neuronal subtypes) is preferable.

Response: Following the suggestion of reviewer, we removed the sentence in the revised manuscript.

- Authors might consider a different nomenclature to refer to vascularized organoids. It is only natural to assume that abbreviations refer to regionalized organoids, so vOrganoids make it sound as "ventral" organoids, which is not what they intend.

Response: We are grateful for the reviewer's comments. We defined the vascularized organoids as vOrganoids in the first place it appears in the revised manuscript.

- The maturation analysis in figure S2 is confusing - choroid should be a separate lineage from excitatory neurons, and not included as a seeming progenitor cell.

Response: We agree with the reviewer that choroid should be a separate lineage from excitatory neurons. Following reviewer's suggestion, the part of maturation analysis was deleted in the revised manuscript,

- In the methods, the co-clustering of scRNA-seq data is poorly explained. For example, what does a union of common highly variable genes mean? Is this the union or the intersection?

Response: We are grateful for the reviewer's comments. Following reviewer's suggestion, in the revised manuscript, we have provided the detailed illustration about the analysis of the scRNA-seq data in the method part of "Mapping organoids cell types to fetal human cortex cell types". (Page 47, Line1078-1087).

References:

Chen, Z., Htay, A., Dos Santos, W., Gillies, G.T., Fillmore, H.L., Sholley, M.M., and Broaddus, W.C. (2009). In vitro angiogenesis by human umbilical vein endothelial cells (HUVEC) induced by three-dimensional co-culture with glioblastoma cells. Journal of neuro-oncology 92, 121-128.

Choudhuri, R., Zhang, H.T., Donnini, S., Ziche, M., and Bicknell, R. (1997). An angiogenic role for the neurokines midkine and pleiotrophin in tumorigenesis. Cancer research 57, 1814-1819.

Daood, M., Tsai, C., Ahdab-Barmada, M., and Watchko, J.F. (2008). ABC transporter (P-gp/ABCB1, MRP1/ABCC1, BCRP/ABCG2) expression in the developing human CNS. Neuropediatrics 39, 211-218.

Daviaud, N., Friedel, R.H., and Zou, H. (2018). Vascularization and Engraftment of Transplanted Human Cerebral Organoids in Mouse Cortex. eNeuro 5.

Fischer, S., Cassivi, S.D., Xavier, A.M., Cardella, J.A., Cutz, E., Edwards, V., Liu, M., and Keshavjee, S. (2000). Cell death in human lung transplantation: apoptosis induction in human lungs during ischemia and after transplantation. Annals of surgery 231, 424-431.

Huber, J.D., Egleton, R.D., and Davis, T.P. (2001). Molecular physiology and pathophysiology of tight junctions in the blood-brain barrier. Trends in neurosciences 24, 719-725.

Hutchison, G.J., Valentine, H.R., Loncaster, J.A., Davidson, S.E., Hunter, R.D., Roberts, S.A., Harris, A.L., Stratford, I.J., Price, P.M., and West, C.M. (2004). Hypoxia-inducible factor 1alpha expression as an intrinsic marker of hypoxia: correlation with tumor oxygen, pimonidazole measurements, and outcome in locally advanced carcinoma of the cervix. Clinical cancer research : an official journal of the American Association for Cancer Research 10, 8405-8412.

Jaffe, E.A., Nachman, R.L., Becker, C.G., and Minick, C.R. (1973). Culture of human endothelial cells derived from umbilical veins. Identification by morphologic and immunologic criteria. The Journal of clinical investigation 52, 2745-2756.

Kuo, Y.C., and Lu, C.H. (2011). Effect of human astrocytes on the characteristics of human brain-microvascular endothelial cells in the blood-brain barrier. Colloids and surfaces B, Biointerfaces 86, 225-231.

Mansour, A.A., Goncalves, J.T., Bloyd, C.W., Li, H., Fernandes, S., Quang, D., Johnston, S., Parylak, S.L., Jin, X., and Gage, F.H. (2018). An in vivo model of functional and vascularized human brain organoids. Nature biotechnology 36, 432-441.

Nakatsu, M.N., Sainson, R.C., Aoto, J.N., Taylor, K.L., Aitkenhead, M., Perez-del-Pulgar, S., Carpenter, P.M., and Hughes, C.C. (2003). Angiogenic sprouting and capillary lumen formation modeled by human umbilical vein endothelial cells (HUVEC) in fibrin gels: the role of fibroblasts and Angiopoietin-1. Microvascular research 66, 102-112.

Okuda, Y., Nakamura, H., Yoshida, K., Enomoto, H., Uyama, H., Hirotani, T., Funamoto, M., Ito, H., Everett, A.D., Hada, T., et al. (2003). Hepatoma-derived growth factor induces tumorigenesis in vivo through both direct angiogenic activity and induction of vascular endothelial growth factor. Cancer science 94, 1034-1041.

Pham, M.T., Pollock, K.M., Rose, M.D., Cary, W.A., Stewart, H.R., Zhou, P., Nolta, J.A., and Waldau, B. (2018). Generation of human vascularized brain organoids. Neuroreport 29, 588-593.

Qian, X., Nguyen, H.N., Song, M.M., Hadiono, C., Ogden, S.C., Hammack, C., Yao, B., Hamersky, G.R., Jacob, F., Zhong, C., et al. (2016). Brain-Region-Specific Organoids Using Mini-bioreactors for Modeling ZIKV Exposure. Cell 165, 1238-1254.

Wekerle, T., Kurtz, J., Sayegh, M., Ito, H., Wells, A., Bensinger, S., Shaffer, J., Turka, L., and Sykes, M. (2001). Peripheral deletion after bone marrow transplantation with costimulatory blockade has features of both activation-induced cell death and passive cell death. Journal of immunology 166, 2311-2316.
