# Supplementary material for: Vascularized human cortical organoids (vOrganoids) model cortical development in vivo
Source: PLoS Biol. 2020 May 13;18(5):e3000705. doi: 10.1371/journal.pbio.3000705 (PMC7250475; doi:10.1371/journal.pbio.3000705)
Supplement: Attachment — Submitted filename: Response to Reviewers 0309_V2.pdf [file pbio.3000705.s013.pdf]

Response to Reviewers

Our Academic Editor advises that the following points are essential for the publication of the paper, whereas the other concerns not listed below are not required.

1) The issue regarding caspase-3 antibody raised by reviewer 2;

The authors demonstrate decreased caspase-3 staining in their vOrganoids compared to controls to support less cell death, but they use an antibody recognizing full length, rather than cleaved, caspase-3. Full length caspase-3 may not accurately reflect apoptotic cell death and measurement of cleaved caspase-3 should be used instead.

Response: We are grateful for the reviewer's suggestions. Following the reviewer's suggestions, we performed the immunofluorescence staining of cleaved CASPASE-3 (CST, Cat #9444S) to detect the cell death.
This assessment was carried out in the cultured organoids and transplated organoids. In the cultured organoids, the ratio of cleaved-CASPASE 3-positive cells in the vOrganoids was 20.9% as compared to 54.8% in the control non-vascularized organoids (Response Fig.1A, B and also new Fig. S1F and G). As for the transplanted organoid grafts, we compared the ratio of cleaved-CASPASE 3-positive cells in the vascularized and non-vascularized organoid grafts at 60 days after transplantation. Consistently, the ratio of cleaved-CASPASE 3-positive cells in the vOrganoid grafts was significantly reduced as compared to that in the non vascularized organoid grafts (Response Fig.2A, B and also new Figure S4A, B). Additionally, we also compared the cell death in organoid grafts (60 dpi) and in the in-vitro cultured vOrganoids at the corresponding days (day 120). It showed that the ratio of cleaved-CASPASE 3-positive cells in the cultured vOrganoids at day 120 were significantly increased compared to the vOrganoid grafts and significantly decreased compared to the nonvascularized organoid grafts (Response Fig.2A, B and also new Figure S4A, B).
Therefore, the results of immunostaining of cleaved CASPASE3 support that there are less apoptotic cell death occurred in the vOrganoids than in the control organoids. And the case was true both for the cultured organoids and for the transplanted organoid grafts. These results were similar to that of CASPASE3. And the statistical analysis were all replaced with the corresponding figures of cleaved CASPASE 3.The related description was also added in the revised manuscript (Page 6-7, Line 161-164; Page 15, Line 382-391).

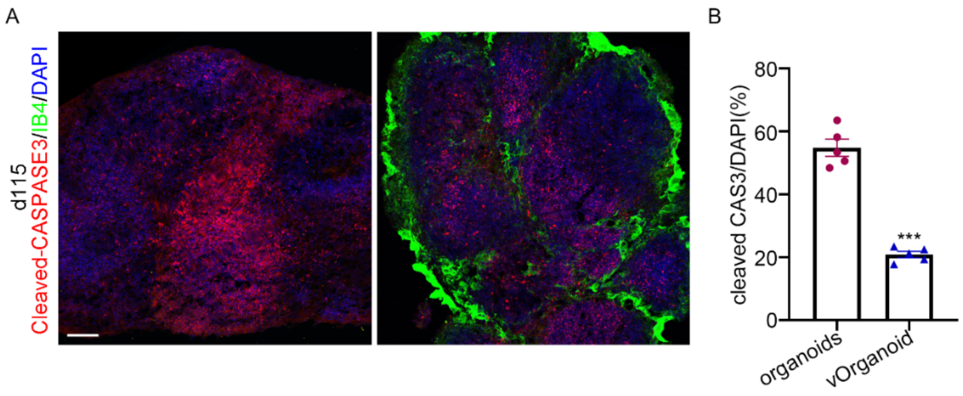

Response Figure 1 Less apoptotic cell death occurred in the vOrganoids than in the control nonvascularized organoids.

(A) Representative immunofluorescence staining figure for cleaved CASPASE 3,IB4 in the control non-vascularized organoids and vOrganoids at d115. Scale bar, 500 µm.

(B) Quantification of the percentages of cleaved CASPASE3+ cells within all cells (DAPI+) in the control organoids and vOrganoids at d115. n=5,5 slices of the control organoids and vOrganoids from three independent experiments. Data are represented as mean ± s.e.m., independent samples t test, *** $p<0.001$.

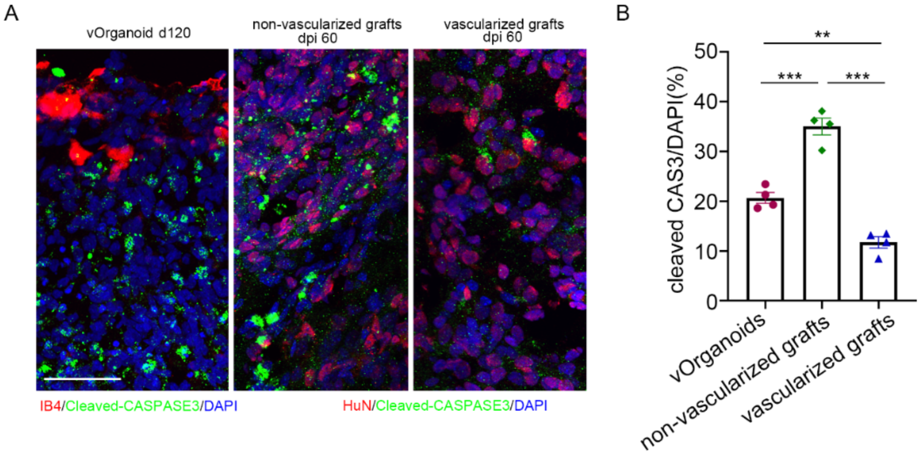

Response Figure 2 Cleaved CASPASE3 immunostaining in non-vascularized and vascularized organoid grafts as well as in the cultured vOrganoids at corresponding days.

(A) Representative immunofluorescence staining figure for cleaved CASPASE 3, IB4 in the cultured vOrganoids at d120 and for cleaved CASPASE3, HUN in the control non-vascularized organoid grafts and the vOrganoid grafts at 60 dpi. Scale bar, 50µm.

(B) Quantification of the percentages of cleaved CASPASE3+ cells within all cells (DAPI+) in the cultured vOrganoids at d120, the non-vascularized and organoid grafts 60dpi, respectively. n=4,4,4 samples for the cultured vOrganoids, the non-vascularized organoid grafts, and vOrganoids grafts from three independent experiments, respectively. Data are represented as mean ± s.e.m., One way ANOVA, ** $p<0.01$, *** $p<0.001$.

2) Reviewer 2's suggestion on citing the new paper by Cakir et al (2019) and all this reviewer's minor concerns;

(i) In the time that the authors revised this manuscript, an additional publication using ETV2-expressing hESCS to generate cerebral organoids with a vascular system was published (Cakir et al, Nature Methods, vol 16, November 2019, 1169-1175). The authors should reference this paper in their introduction and discuss/compare this paper with the present results in the conclusion of this manuscript.

Response: We thank the reviewer for pointing this out. In the revised manuscript, we cited the paper of Cakir et al,2019 in the introduction (Page 4, Line100-102) and discussed their results in the discussion (Page17, Line 439-442; Page20, Line507-510).

(ii) Additional minor concerns of reviewer 2:

(1)Several abbreviations (e.g. vRG, oRG) remain undefined in the text.

Response: We thank the reviewer for pointing this out. We carefully looked over and defined all the abbreviations at the first places that they appeared in the revised manuscript.

(2)The authors responded that they toned down the claims of lamination in the organoids, but in the abstract, they still state that their model recapitulates lamination of the neocortex. This statement should be removed.

Response: Following the reviewer's suggestion, we removed the statement of organoids recapitulates the lamination of the neocortex in the abstract of the revised version.

(3)In lines 100-101, the authors state that "all of these studies indicate that vascularization is important for organoid survival." This is overstated, as there are plenty of protocols that do not have vascularization but have good organoid viability.

Response: We are grateful for the reviewer's comments. In the revised manuscript, we revised this statement as "All of these studies indicate that vascularization is one of the feasible methods for improving organoid survival" (Page 4, Line108-109).

(4)For Fig S1G, it would be preferable if the data were presented as individual data points with mean/standard deviation rather than a bar graph, to better demonstrate the variability/range of the data.

Response: We are grateful for the reviewer's suggestions. And following the suggestion, we presented the data in Fig.S1G as individual data points with mean/s.e.m.. (Response Figure 3 and also new Fig. S1G).

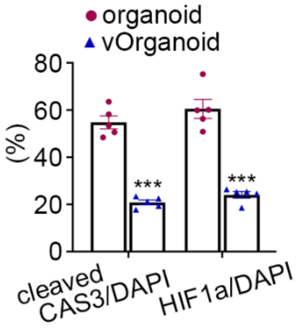

Response Figure 3 Quantification of the percentages of cleaved CASPASE3+ cells (left) and HIF1α+ cells (right) within all cells (DAPI+) in the control organoids and vOrganoids at d115, respectively.

For cleaved CASPASE 3, n=5,5 slices of the control organoids and vOrganoids from three independent experiments. Data are represented as mean ± s.e.m., independent samples t test, *** p<0.001. For HIF1α, n=5, 5

slices of control and vOrganoids in three independent experiments. ata are represented as mean±s.e.m., independent samples t test, *** p<0.001.

(5)The claim that vOrganoids are larger than controls (Fig. S1H) is questionable. The error bars appear very small compared to what might be expected for typical size variability of organoids--are the error bars standard deviation or standard error of the mean? Which statistical test was performed? It would be appropriate to do 2-way ANOVA with multiple comparisons, rather than do repeated t-tests. Even if the data are statistically significant, the relative difference in size does not appear to be of biological significance.

Response: For the old Fig.S1H, The data were presented as mean ± s.e.m.. And the statistical test used was t-test. Following the reviewer's suggestion, we did 2-way ANOVA for the data. The results were shown in response figure 4 and new Fig.S1H.

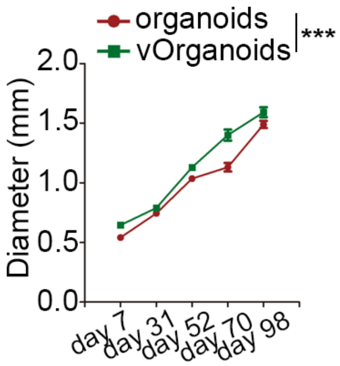

Response Figure 4 2-way ANOVA analysis of the size of the control organoids and vOrganoids that generated from H9.

n=11,11,11,11,11 for day 7, day 31, day52, d70, and day98, respectively. Data are represented as mean ± s.e.m., two way ANOVA analysis, *** p<0.001.

(6)For Fig. S1L, there is no control staining—perhaps the HUVECs express p-gp even in the absence of being cultured with the organoid?

Response: We are grateful for the comments. Following the reviewer's suggestions, we added a control staining. First, HUVECs were under an *in vitro* matrigel angiogenesis assay to form tube-like structures. Then, the HUVECs tube-like structures were immunostained for IB4 and p-gp, showing that the tube-like structures were immuno-positive for IB4 and immuno-negative for p-gp. These results indicate that when being cultured individually without organoids, HUVECs could form tube-like structures, which were similar to those in vOrganoids but did not express p-gp (Response Figure 5 and also new Fig. S1M). Methods and related results were also updated (Page 7, Line172-185; Page 38, Line 1150-1162).

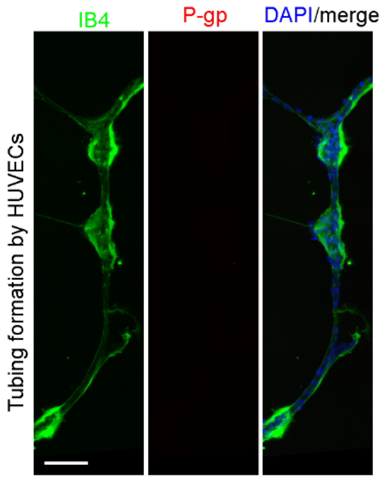

Response Figure 5 Immunofluorescence staining for p-gp/IB4 in the tube-like structure formed by HUVECs. Scale bar,100um.

(7)The calcium imaging data in Fig 3I was normalized to 1 for both the d50 and d85 conditions. In order to see relative differences between d50 and d85 (one would expect increased activity with older organoids), the data should be normalized to just the day 50 organoid in ACSF.

Response:

We thank for the reviewer's suggestion. We normalized relative Ca2+ transients frequency with the ACSF data of day 50. (n = 188, 124; 31, 9; 65, 33; 77, 36; 15, 46. p = 0.1266, 0.2798, 0.0208, 0.00005 and 0.0075 for ACSF, TTX, CNQX, APV and BMI groups. Data are represented as mean ± s.e.m., two-sample t-test, *p<0.05, **p<0.01, ***p<0.001, ****p<0.0001). The mean activity of ACSF control is slightly higher in day 85 but not significant. (Response Fig.6 and also new Fig.3I)

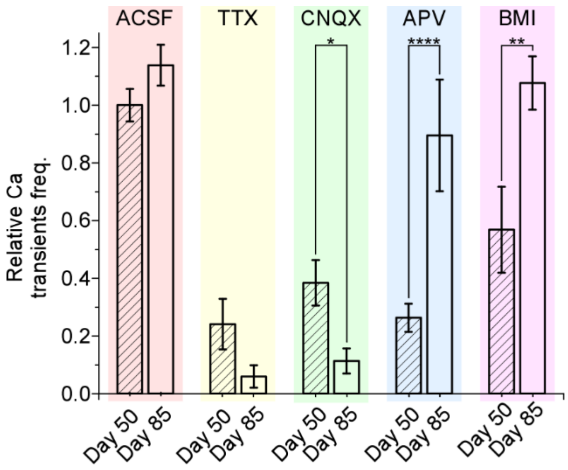

Response Figure 6 The effects of different treatments on spontaneous calcium fluctuations were compared between day 50 and day 85 in the statistical results.

(8)The authors should provide additional explanation regarding the response of calcium transients to pharmacological treatments of the organoids (Fig. 3I and lines 274-281). Why are CNQX and APV more effective at reducing calcium transients at the later time point? The authors should also explain in more detail the effect of BMI. The effect at d50 of decreasing calcium transients may be a developmental effect, where opening of GABA-A receptors can depolarize the cell membrane, since the chloride ion concentration is higher inside the cell compared to outside the cell. The effect at d85 (where there does not seem to be an effect of the BMI), may be the transition point when the intracellular chloride ion concentration is decreasing, and opening of the GABA-A receptors may not have an effect on the membrane potential.

Response: We thank reviewer for this critical comment. We observed a reduced sensitivity to APV and greater sensitivity to CNQX at day 85, which indicated a transition from NMDA receptor- to AMPA receptor-mediated excitatory synaptic activities during the maturation of the vOrganoids. This is similar to what happens in vivo, as previously reported (Yu et al., 2014). The effect of BMI may be induced by the decreasing intracellular chloride ion concentration which is due to the developmental expression of KCC2 (Rivera et al., 1999). We added this explaination in the revised manuscript (Page13-14, Line333-348).

(9)Figure S3H and I are discordant with each other. Why do the tracings in the ACSF condition in panel H show calcium transients, but the ACSF in panel I has even less activity than the amount seen in the TTX condition of panel H?

Response: We thank reviewer for this critical comment. To identify the effect of TTX which blocked the action potential of cells, we chose active cells which exhibited several calcium transients to start with as examples in new Fig. S3N. Glutamate is a neurotransmitter to excite the neural activity, so we chose cells which barely had calcium transients before drug application as examples to show the stimulating effects of glutamate (new Fig. S3O) .

(10)For Figure 4C, one cannot conclude that there is synaptic connectivity between the organoid graft and the host brain. The co-localization of pre-synaptic and post-synaptic markers could also be mouse-to-mouse or human-to-human.

Response: We are grateful for the comments. We agree with the reviewer that just by the results of the immunofluorescence staining of SYB2 and PSD95 could not conclude the presence of synaptic connectivity between the organoid graft and the host brain. In the revised manuscript, we revised our conclusion by only stating that there is synaptic connectivity in the grafts.The pre-synaptic and post-synaptic sources could not be distinguished in the current studies (Page15, Line376-381).

3) Reviewer 3's concern on Figure 2K and Table S1 - most part of this reviewer's concern #2;

(i) The study states scRNA-seq suggests improvement in gene expression and neurogenesis in vOrganoids.

I'm particularly concerned about the scRNA-seq data analysis. The interpretation of clustering is useful and very interesting that subcortical cell types emerge. However, these datasets could allow for a quantification of whether vOrganoid cells of particular types more closely resemble fetal cell counterparts than control organoids. Instead, the study simply implies the vOrganoid cells are improved, but only shows that you can integrate the organoid cells with primary cells using a batch correction algorithm. However, these batch correction algorithms can overcome severe differences between conditions, and it is unclear whether the integration is superior with vOrganoid cells. Figure 2K could further help address if vOrganoids bring cells closer to fetal cell gene expression if the differential expression between vOrganoids were put in context of fetal cell data.

Response: We thank for the reviewer's suggestion. Based on the analyses of scRNA-seq, we did not conclude that the cell types of vOrganoids were more similar to the the human fetal cell counterparts than the controls in our manuscript. Firstly, the cell types of vOrganoids and controls was determined based on the expression of known marker genes in the Fig.2A and Fig.S2C (Response Fig.7, also Fig.2A and Fig.S2C). Then,we calculated the transcriptomic correlation matrix between the cell types of the vOrganoids and controls. The high transcriptomic correlations between the corresponding cell types of the control organoids and vOrganoids suggested that there are no remarkable cell type differences between the non-vascularized organoids and vOrganoids.

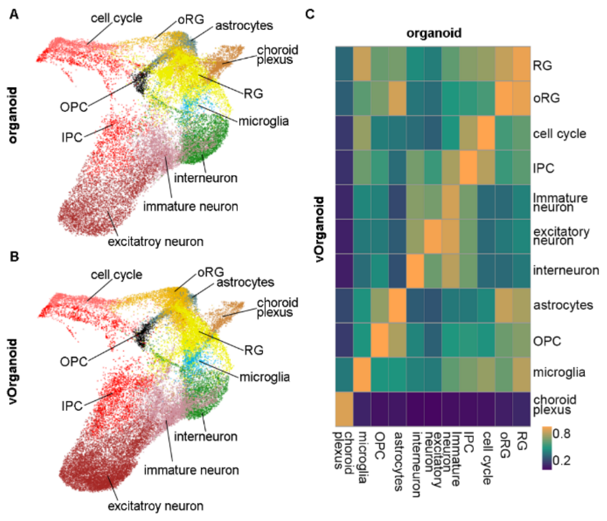

Response Figure 7 No remarkable cell type differences between the non-vascularized organoids and vOrganoids were detected.

(A) Visualization of the major cell types in the control organoids by three dimensional UMAP. Each dot represents one individual cell and colored by cell types.

(B) Visualization of the major cell types in vOrganoids by three dimensional UMAP. Each dot represents one individual cell and colored by cell types.

(C) The transcriptomic correlations between the cell types of control organoids and vOrganoid were visualized via heatmap (correlation coefficient: yellow, high; purple, low).

Secondly, in order to eliminate the potential effects of batch correction algorithm on the integration of cells of vOrganoids/control organoids with human fetal cells and to understand whether the cell types of vOrganoid were in higher similarity to the fetal cell counterparts than the controls, we separately integrated the control organoid or vOrganoid cells with human fetal cells (Response Fig. 8 and new Fig.S2G-N). Hence, we got two individual datasets, one is the control organoid and human fetal cells (Response Fig. 8A-B and new Fig.S2G-H) and the other is the vOrganoids and human fetal cells (Response Fig. 8 C-D and new Fig.S2I-J). Next, we generated the PAGA graphs to show the relationships between the cell types of control orgnaoids/vOrganoids and human fetal cells, respectively (Response Fig.8E-F and new Fig.S2K-L). In the PAGA network, the cell types of control organoid as well as that of vOrganoid both highly correlated with the corresponding ones in the human fetal telencephalon (Response Fig.8E-F and new Fig.S2K-L). Besides, we also computed the transcriptomic correlation matrix between the cell types from two different sources. It showed that the cell types of control organoids or vOrganoids all showed comparable high correlations to the corresponding cell types of human telencephalon (Response Fig.8G-H and new Figures S2M-N). Collectively, these analyses suggested that the cell types of vOrganoids do not exhibit higher similarity to the human fetal cell counterparts than the control organoids.

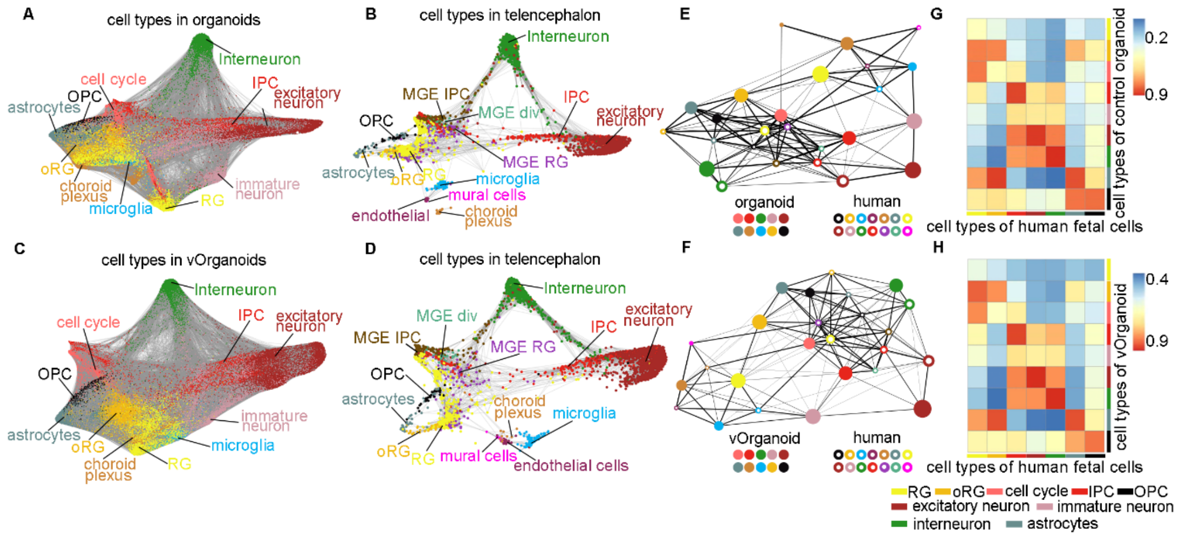

Response Figure 8 Analysis to evaluate the similarity of cells in the control organoids/vOrganoids to the human fetal cells.

(A-B) After being integrated, the cell distributions of control organoids (A) and human fetal telencephalon (B) were visualized in forced-directed graph separately. Each dot represents a single cell and is colored according to

the cell types. Width of edges is scaled with the cell-cell connectivity.
(C-D) After being integrated, the cell distributions of vOrganoids (C) and human fetal telencephalon (D) were visualized in forced-directed graph separately. Each dot represents a single cell and is colored according to the cell types. Width of edges is scaled with the cell-cell connectivity.
(E-F) The correlations between the cell types of control organoids and human fetal telencephalon (E) as well as that between the cell types of vOrganoids and human fetal telencephalon (F) are displayed in the PAGA graph. The cell types in the organoids and human fetal cortex datasets were denoted by the solid and hollow dots, respectively. And the same cell types in two datasets were colored identically. The size of dots was scaled with the cell numbers; width of edges was scaled with the connectivity between cell types.
(G-H) The transcriptomic correlations between the cell types of control organoids (G) or vOrganoids (H) and human fetal telencephalon were visualized via heatmap. The correlation coefficient values were labeled out (correlation coefficient: red, high;blue, low).

Next, we asked what are the differences between the vOrganoids and the control organoids.We compared the development progress of vOrganoids and control organoids by analysing the scRNA-seq data via two different methods. Firstly, we predicted the developmental states of the cells in the control organoids and vOrganoids along the pseudotime trajectory by monocle analysis(Cao et al., 2019; Qiu et al., 2017; Trapnell et al., 2014). The fitted curves of cell density along the pseudotime were plotted. Both at day 65 and day 100, more vOrganoid cells were assigned in high pseudotime score while fewer cells were under low score as compared to the control organoids (Response Fig.9A and also new Fig. 2F). However, the extent of divergence was much more obvious at day65 than at day100 (Response Fig.9A and also new Fig. 2F). These results indicated that vOrganoids developed much faster than the controls at early stages, even though this difference was decreased along the development. Secondly, we also ordered the cells of vOrganoids and controls along the maturation trajectory by calculating the maturation score. The fitted curves of cell density along the maturation trajectory were plotted. And similar to the results of pseudotime, there are fewer vOrganoid cells in the low maturation scores and more in the high scores than the control organoids (Response Fig.9B and new Fig. 2G). Besides, the differences were more distinct at day65 than at day100 (Response Fig.9B and new Fig. 2G). Collectively, both the pseudotime and maturation trajectatory analysis suggested that the vascular systems in the vOrganoids might accelerate the development of vOrganoids at the early stage.

Then, to understand why vOrganoids develop faster than control organoids, we analyzed the cell composition at day 65 (Response Fig.10 and also new Fig.S2O). It showed that the percentage of one type of neural progenitor cells – oRG were much higher in vOrganoids than in controls (Response Fig.10 and also new Fig.S2O). oRG cells are originated from RG and play important roles in the neurogenesis, which greatly contributing to the rapid excitatory neurons production during the human fetal cortical development (Hansen et al., 2010; Wang et al., 2011). Accordingly, the progenies of neural progenitor cells -the excitatory neuron percentage in vOrganoids was higher than in control organoids at day 65 (Response Fig.10 and also new Fig.S2O). Thus, associated with the results of immunostaining analyses of NEUROD2+ excitatory neurons (new

Fig.2H-I), the enhanced ratio of oRG and excitatory neurons in the scRNA seq data of vOrganoids suggested that the vascular systems in vOrganoids may promote neurogenesis in vitro. The enhanced neurogenesis may account for the accelerated development in vOrganoids.

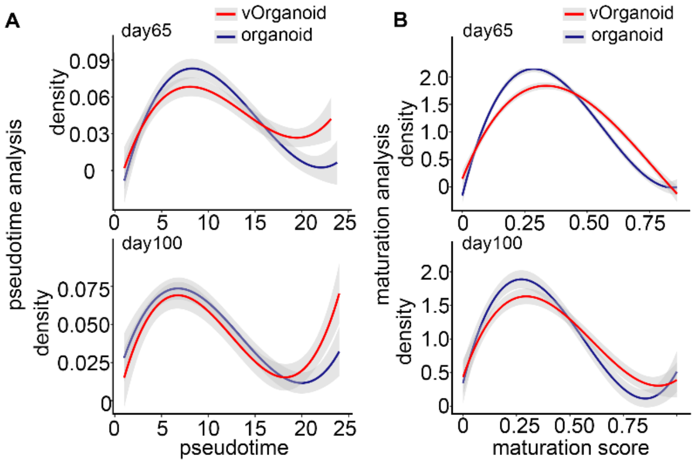

Response Figure 9 vOrganoids are in a more mature developmental state as compared to the control organoids

(A) The fitted curves of cell density along the pseudotime at day 65 (upper) and day 100 (lower) were visualized.

(B) The fitted curves of cell density along the pseudo-maturation trajectory at day 65 (upper) and day 100 (lower) were visualized.

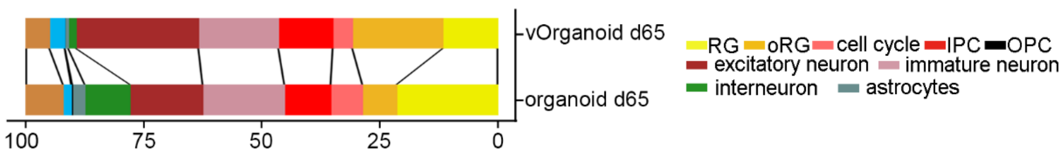

Response Figure 10 The composition of cell types in the control organoids and vOrganoids at day 65 was illustrated in the bar graph.

We agree with the reviewer that these further analyses will provide more information than Figure 2K. So we added these figures into the the new Figure 2 and new Figure S2.

(ii) However, I have major reservations with this analysis and with Table S1. First, it is unclear what cell types are listed in the Fig 2K table. Is there a cell cycle gene expression difference between progenitors across vOrganoid and control conditions, or are there more progenitor cells in the control organoids? A meaningful comparison must analyze cells of the same type, but the heatmap looks like some sort of composition difference between the control and vOrganoid cells analyzed, with more dividing progenitors in the control organoids.

In this regard, the enrichments in Figure 2K and Table S1 are incomplete, unconvincing, and potentially misleading. The enrichments of vOrganoid cells in "axon guidance," "blood vessel morphogenesis" and "cellular response to oxygen levels" sound at first like they represent a more fetal-like state (and a quick read of the manuscript would interpret this analysis as evidence of improvements in vOrganoids, but the genes listed in the table for enrichments are either very few (3 genes in total account for multiple statements about cellular response to oxygen!) or indicative of an artifact (6/7 genes listed for axon guidance are ribosomal protein transcripts that often reflect outlier cells to be removed in scRNA-seq QC!). The p-values for the GO terms also must be corrected for multiple hypothesis testing.

This analysis should be dropped or completely re-done, explicitly addressing how cells of the same type differ across conditions and whether these differences produce more fetal-like cells in vOrganoids.

Response：We are grateful for the comments. The scRNA-seq data involved in the studies were firstly proceeded to under quality control procedures based on the following criterion: nFeature_RNA > 200 or nFeature_RNA < 6000, mitochondrial gene percentage < 10%. Cells with percentage of haemoglobin reads > 1% (‘HBA1’, ‘HBA2’, ‘HBB’, ‘HBD’, ‘HBE1’, ‘HBG1’, ‘HBG2’, ‘HBM’, ‘HBM’, ‘HBQ1’, ‘HBZ’) were discarded as well. In total 57180 cells remained for subsequent analysis. After these treatments, the cells and genes with poor qulity were excluded in the subsequent analysis.

We applogize for not providing information clearly. The cell types listed in the old Fig.2K were the IPCs and the immature and excitatory neurons. With reviwer’s comments, we carfully thought about this analysis and we agree that the cell types were not well selected. So, we re-did the analysis in the revised manuscript. The analyses about cell type (Response Figure 7, 8), developmental stages (Response Figure 9) and cell proportion (Response Figure 10) were listed in the previous answers. From these analysis, we did not see the cell type differences, but we observed the cell proportion differences. These analyses indicated that the differences between vOrganoids and controls might come from progenitors (Response Fig. 9, 10). What’s more, the intimate interactions between the vasculature and proliferative progenitor cells during brain development(Androutsellis-Theotokis et al., 2009; Jin et al., 2002; Kazanis et al., 2010; Sun et al., 2006) prompts us to explore whether the vascular systems in vOrganoids could influence the gene expression in the progenitors. Hence, we checked the gene expression difference between the progenitors (including RG, oRG, IPC, and cell cycle active cells at day 65) across vOrganoid and control conditions. The enriched gene ontology of the DEGs and the adjusted p values were listed (Response Fig. 11 and also new Figure 2J). The GO terms enriched in the progenitor cells of control oraganoids were response to hypoxia, positive regulation of cell death (Response Fig. 11 and also new Figure 2J).These GO terms are consistent with the observation that more HIF1a+ and cleaved CASPASE3+ cells in the nonvascularized organoids (Figure S1F). Meanwhile, the GO terms enriched in the progenitor cells of vOrganoids were positive regulation of neurogenesis, blood vessel morphogenesis (Response Fig. 11 and also new Figure 2J), which accounts for the vasculature formation, as well as improved neurogenesis in vOrganoids. According to the reviewer’s suggestion, the p values for the GO

terms were corrected for multiple hypothesis testing. Correspondingly, the Table S1 were replaced with the GO terms and related DEGs of progenitor cells in vOrganoids and control organoids.

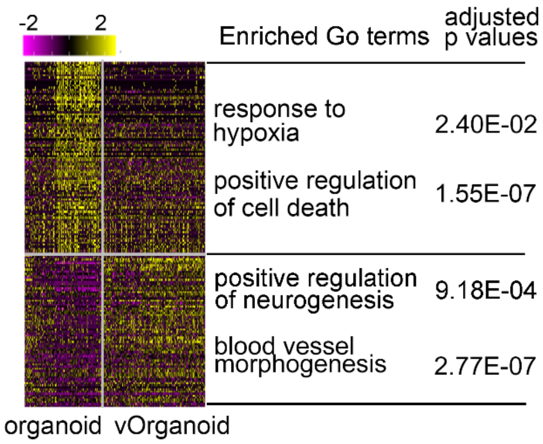

Response Figure 11 Heatmap showing the expression of DEGs between the progenitor cells of control organoids and vOrganoids at day 65. The enriched gene ontology of the DEGs and the adjusted p values are also listed.

4) Reviewer 3's concern #3. Specifically, "clearly state what steps were taken to record from neurons of the same type in an unbiased way because differences could arise from recording from different neuron subtypes between conditions, or even neurons of different size. Is the result of a difference between vOrganoid and control organoids significant, or just a trend, no p-value is provided?";

Response: We thank reviewer for this critical comment. In order to unbiasedly record from neurons of the same type in vOrganoids and the control organoids, we selected the neurons with similar soma diameters and located within 150 μm away from the edge of the organoid slices. And the statistical results showed that neither the soma size (Response Fig. 12A and new Fig. S3A) nor the soma location (Response Fig. 12B and new Fig. S3B) of patched cells has significant difference between organoids and vOrganoids. These results suggest that we selected cells to record in an unbiased way for organoids and vOrganoids.

We further analyzed the cell capacitance (Response Fig.12C and new Fig.S3G), the resting membrane potential (Response Fig.12D and new Fig.S3F) and cell firing ability under different amplitudes of injected currents (Response Figure 12E-H and new Fig.S3D-E). There were significant differences in cell capacitance between organoids and vOrganoids in day 80 and day 90 (Response Fig.12C and new Fig.S3G). This result suggests that with comparable soma size, vOrganoid cells have more membrane surface area for dendrites or axon growth. Besides, the vOrganoids cells reached a more hyperpolarized membrane potential than organoids cells in day 80 (Response Fig.12D and new Fig.S3F). Furthermore, more cells in vOrganoids were firing under a current stimulation than cells in organoids at day 80 and day 90 (Response Fig. 12F-H and new Fig.S3D-E). P-values were calculated and illustrated in the figure legends.

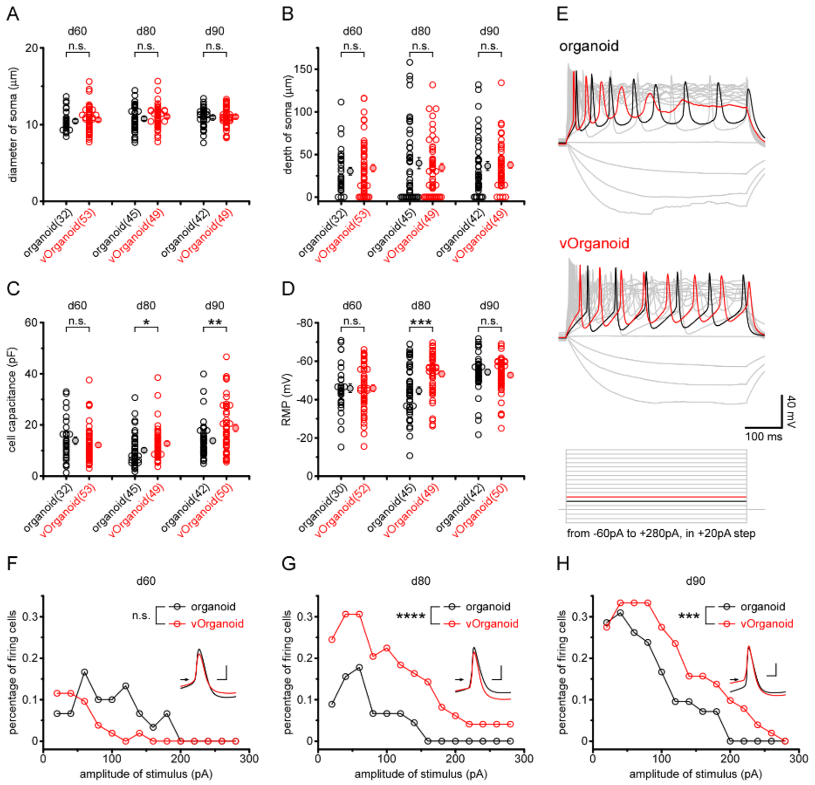

Response Figure 12 Electrophysiological properties of cells from organoids and vOrganoids

(A) Diameters of somata of recorded cells from organoids and vOrganoids at day 60, day 80 and day 90. p = 0.6026, 0.3494 and 0.7491 for day 60, day 80 and day 90, two-sample t-test. (A-D): Open circles indicate data from individual cells. Filled circles indicate the mean value. Data are represented as mean ± s.e.m., two-sample t-test, *p<0.05, **p<0.01, ***p<0.001.

(B) Depth of somata of recorded cells from organoids and vOrganoids at day 60, day 80 and day 90. p = 0.5799, 0.5248 and 0.8651 for day 60, day 80 and day 90.

(C) Capacitance of recorded cells from organoids and vOrganoids at day 60, day 80 and day 90. p= 0.3059, 0.0424 and 0.0075 for day 60, day 80 and day 90.

(D) Resting membrane potentials of recorded cells from organoids and vOrganoids at day 60, day 80 and day 90. p = 0.9842, 0.00059 and 0.4102 for day 60, day 80 and day 90.

(E) Representative evoked action potentials of cells from organoid and vOrganoid at day 90. The amplitudes of injected currents are from -60pA to 280pA in a step of 20pA.

(F-H) The percentage of firing cells under currents stimulation with different amplitudes. The insertion indicates the evoked action potential of cells from organoids (black) and vOrganoids (red). The scale bars indicate 5ms and 20mV. The arrow indicates -20mV. p = 0.0602, 0.0000025 and 0.00012 for day 60, day 80 and day 90. Data are represented as mean ± s.e.m., paired-sample t-test, *p<0.05, **p<0.01, ***p<0.001, ****p<0.0001.

5) Part of reviewer 3's concern #4 : "a control experiment of whether induction occurs in HUVECs cultured on their own, and probably a control experiment that the P-glycoprotein antibody labels human HBMECs";

Response: We thank the reviewer for pointing this out. Following the reviewer's suggestions, we added two control experiments. First, we performed the immunostaining of IB4 and p-gp in GW12 human cortex slices to check whether the P-glycoprotein expressed by human HBMECs. The results showed that the human HBMECs could be co-labelled by IB4 and P-gp, indicating that the P-glycoprotein antibody could faithfully label human HBMECs (Response Fig.13A and also new Fig.S1L). Second, we performed the immunostaining of IB4 and P-gp in tube-like structures formed by culturing HUVECs alone in matrigel (Response Fig.13B, and also new Fig.S1M). The results showed that the tube-like structures were immuno-positive for IB4 and immuno-negative for P-gp, indicating that when being cultured individually without organoids, the induction of HUVECs to transform to brain like endothelial cells do not occur.The related description was added in the revised manuscript (Page7, Line172-185).

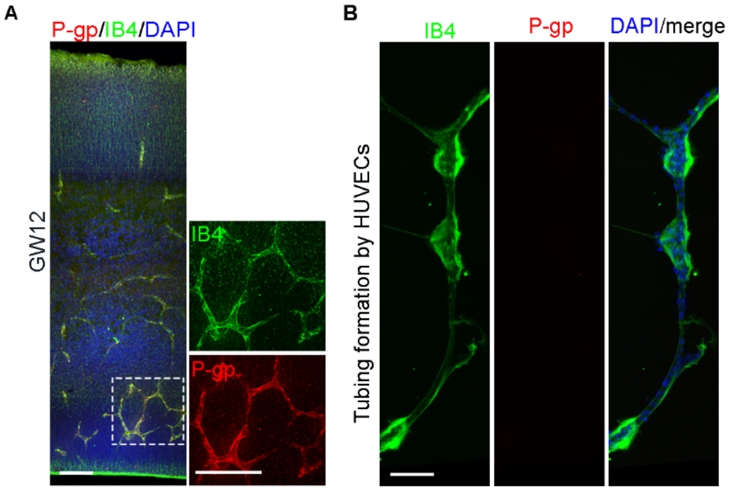

Response Figure 13 Immunostaining for IB4 and P-gp in the human brain slice (A) and tube-like structures formed by HUVECs (B).

(A) Immunostaining for P-gp and IB4 in the human cortial slices at GW12. Scale bar, 100um.

(B) Immunostaining for P-gp and IB4 in the tube-like structure formed by HUVECs. Scale bar, 100um.

6) All the minor concerns from reviewer 3.

(i) abstract still says "recapitulate lamination of neocortex" but there is no evidence for cortical layers resembling in vivo lamination.

Response: We thank the reviewer for pointing this out. In the revised manuscript, we removed the description of "recapitulate lamination of neocortex" in the abstract.

(ii) The data in figure panels does not support the text that "later-born superficial layer neurons (SATB2+cells) were more superficially localized to the CTIP2+ neurons" - certainly the SATB2 neurons accumulate superficial to SOX2 and TBR2 but evidence for cortical plate lamination is weak in organoid field beyond some coarse SATB2 CTIP2 stains that lack quantification across replicates, individuals, and developmental time. Even the TBR1 staining in S1O extends to the surface and there is RELN staining

very interior. Regardless, without comparing quantitative cortical plate lamination phenotypes in vOrganoids to control organoids, it is impossible to know whether the resolution of layers improves by this treatment. In addition, while it is true that virtually all upper layer projection neurons are SATB2+, over 30% of deep layer neurons project through the corpus callosum and are SATB2+ (refer to McConell, Chen, Macklis, and Arlotta labs literature). I would encourage the author to either refer to these cells as callosal neurons or use markers with a more clear laminar restriction such as CUX1 and CUX2 (for example Quadrato et al.)

Response: Following the reviewer's suggestions, we changed our statements as "Furthermore, we found that some SATB2+ cells were superficially localized to CTIP2+ neurons and some were widely distributed in the migrating zone and newly formed cortical plates in vOrganoids (Figure 1H, right panel; 1I, left panel; and S1P). This observation was consistent with reports showing that SATB2 is expressed by virtually all upper layer projection neurons as well as 30% of deep layer neuron that project through the corpus callosum (Alcamo et al., 2008; Britanova et al., 2008; Leone et al., 2015)" to tone down the statement of lamination (Page 8, line191-196). We have also shown the quantification of the percentage of SATB2+, BRN2+ and CTIP2+ cells in the vOrganoids of day 128 and day 210, respectively (Figure 1M). The quantification was based on from three independent experiments (3 organoids for each independent experiment). All data are presented as means ± s.e.m., independent-samples t test, $*p < 0.05$, $**p < 0.01$.

(iii) Figure S1 E show less extensive IB4 staining in iPSC-derived organoids and Figure S1F shows much of the IB4 localization on the surface of older organoids (D115), which differs from the internal staining in Figure 1G (d45, d65). From the discussion, it sounds like the vasculature being precisely in the VZ/SVZ during early culture stages - can the authors clarify how the HUVEC distribution changes over time and how consistent the transition is?

Response: We thank the reviewer for the comments. In the early culture stage, the vascular structures preferred to be adjacent to neural progenitors and were located precisely above the VZ/SVZ region (day 45) (Fig.1D, E), while some were also detected in the migrating zone and cortical plate, which is consistent with the location of the blood vessels in vivo (new Fig.S1C). However, from day 65 to day 200, more and more neurons were generated and the migrating zone and cortical plate expanded while the VZ/SVZ area shrunk. During this process, the vascular structures gradually emerged in the migrating zone and cortical plate. This distribution trends were consistent in vOrganoids. We added the description and discussion in the revised manuscript (Page6, Line145-153; Page 18, Line 451-455).

(iv) In Fig 2 - why use both UMAP and PAGA for visualization of dimensionality reduction?

Response: We are grateful for the comments. UMAP (Uniform Manifold Approximation and Projection) is a commonly used algorithm for dimension reduction and could preserve data's global structure. PAGA (Partition-

based Graph Abstraction), an approach developed by Alexander Wolf et al(Wolf et al., 2019), is able to reconstruct the biological lineage relations based on computing the connectivities between partitions (annotated cell clusteres) besides visualization of dimension reduction.

In our work, we generated UMAP graph for visualizing features of cells (such as cell annotation, gene expression). PAGA graph was computed to investigate whether cell types of orgnaoids could greatly resemble that of fetal human telencephalon as it allows us to obtain the celltype-celltype relations. The PAGA graph delivered us information about the cell type correspondences between orgnaoid and fetal human cells.

(v) Also, explain what PAGA stands for and how it is performed.

Response: PAGA stands for Partition-based Graph Abstraction, an approach developed by Alexander Wolf et al(Wolf et al., 2019). To perform PAGA, we first constructed AnnData data structure with the integrated ('batch-corrected') gene expression matrix (the gene expression matrix contained both orgnaoids and human fetal telencephalon cells) generated through the Seurat v3 datasets integration procedure. Next, we did PCA dimension reduction analysis with function sc.tl.pca, computed the neighborhood graph with function sc.pp.neighbors and constructed the PAGA graph with function sc.tl.paga. The PAGA graph was plotted with function sc.pl.paga. This information was updated in Methods (page 44-45, Line 1322-1327). The full name of PAGA was given at the firt place it appeared in the manuscript (page 9, Line227-229).

(vi) In Figure 2, explain the method for batch integration and how it was corroborated by other methods.

Response: Our organoid dataset and the published human fetal telencephalon datasets were sequenced by different platform 10x and C1, respectively. And the batch integration of Seurat aims to identify shared cell states acorss different datasets produced under different experimental conditions and then to integrate these datasets. And in previous studies, the batch intergration of Seurat was also widely used in the scRNA-seq data from different platforms or from different samples (Durante et al., 2020; Kim et al., 2019; Mancuso et al., 2019; Soliman et al., 2020; Wen et al., 2020). Thus, the Seurat integration approach (Seurat V3) is appropriate and been chose to integrate our organoid dataset with the published human fetal telencepalon dataset.

For the integration, we first identified highly variable genes of each dataset using Seurat function FindVariableFeatures and ranked them by examining the number of datasets in which they were independently identified as highly variable genes. We then passed the top 2000 genes to the function FindIntegratoinAnchors to identify correspondences among these two datasets. Then, we run function IntegrateData to compute a batch corrected gene expression matrix that contains all cells from both datasets. The resulted integrated matrix was then used for downstream dimension reduction analysis. This information was updated in Methods (page 44, Line 1310-1321).

(vii) What is the physiological significance of increased outward current in vOrganoids and what other properties might be expected to change along with this one?

Response: We thank reviewer for this critical comment. We analyzed the firing ability of organoids cells and vOrganoids cells under a series of currents stimulations (Response Fig.12E and also new Fig.S3D). More vOrganoids cells exhibited firing responses than organoids cells under a same current stimulation from day 80 (Response Fig. 12G-H and also new Fig.S3E) when vOrganoids cells obtained a increased outward current. And vOrganoids cells can respond to a strong stimulation which organoids cells did not respond to (Response Fig.12 G, >160pA in day 80; Response Fig.12H, >200pA in day 90). Given that the outward current produces the repolarizing phase of the action potential and contributes to the cell's firing response to injected currents, the increased outward currents might in turn contribute to the higher firing ability under currents stimulation with different amplitudes in vOrganoids.

(viii) it is uncertain whether there is synaptic connectivity between the mouse brain and organoid cells based on these studies, although other reports (Mansour, Vanderhagen, de Paola indicate this occurs)

Response: We are grateful for the comments. We agree with the reviewer that just by the results of the immunofluorescence staining of SYB2 and PSD95 could not conclude the presence of synaptic connectivity between the organoid graft and the host brain. In the revised manuscript, we restated our conclusion as " The colocalization and close association of SYB2 and PSD95 indicated that there was synaptic connectivity in the grafts (Figure 4C). However, the presynaptic and postsynaptic sources could not be distinguished in the current study." (Page15, line378-381)

(ix) It is very interesting that "the vascular systems in the vOrganoids could improve the cell survival in the host" - Fig 4E is exciting, providing evidence for the host vasculature connecting to the HUVECs, which could explain the improvements in transplant of vOrganoids

-This study observes a 40% Caspase3/DAPI staining in grafted organoids that lack HUVEC cells, which seems very high compared to Mansour et al 2018 who observe almost no TUNEL staining in day 50 and day 233 post-impantation organoids - is there an interpretation for this different observation? The Mansour paper makes it sound like grafting a normal organoid already solves the apoptosis problem, but that does not seem to be the case here. Because the improved grafting of vOrganoids may be a key feature, it would be important to address this discrepancy in some way, even just in the text.

Response: We are grateful for the comments.One of the reviewer pointed out that cleaved caspase3 was more feasible than caspase 3 for illustrating the apoptotic cell death in organoids.And following this suggestion, we performed the immunostaining for cleaved caspase 3 and replaced the pictures in the Fig.S4A and B (new Fig.S4A and B). We guess the discrepancy could be due to the differences between culture and transcplantation methods, transplantation location (retrosplenial cortex vs. S1 cortex) and cell death quantification methods (cleaved caspase3 staining vs. TUNEL assay). We add related discussion of this point in the revised manuscript (Page19-20, Line499-507).

(x) Figure 4B organoid staining does not resemble cortical plate lamination or cortical folding. There are

more clusters of SATB2 by the surface, but these do not form a layer, and as acknowledge, these are interrupted by VZ-like rosettes, which would account for the bumpiness described as cortical folding. Strong claims of cortical layer formation and cortical folding require strong evidence, including clear definitions and quantification which are lacking, and it is unhelpful for the field to promote confusion here. Also, it is unclear how these observations compare to control organoids.

Response: We are grateful for the comments. Following the reviewer's suggestions,we removed the statement of cortical plate lamination and cortical folding in the revised manuscript as for Fig.4B.

Reference

Alcamo, E.A., Chirivella, L., Dautzenberg, M., Dobreva, G., Farinas, I., Grosschedl, R., and McConnell, S.K. (2008). Satb2 regulates callosal projection neuron identity in the developing cerebral cortex. Neuron 57, 364-377.

Androutsellis-Theotokis, A., Rueger, M.A., Park, D.M., Mkhikian, H., Korb, E., Poser, S.W., Walbridge, S., Munasinghe, J., Koretsky, A.P., Lonser, R.R., et al. (2009). Targeting neural precursors in the adult brain rescues injured dopamine neurons. Proceedings of the National Academy of Sciences of the United States of America 106, 13570-13575.

Britanova, O., de Juan Romero, C., Cheung, A., Kwan, K.Y., Schwark, M., Gyorgy, A., Vogel, T., Akopov, S., Mitkovski, M., Agoston, D., et al. (2008). Satb2 is a postmitotic determinant for upper-layer neuron specification in the neocortex. Neuron 57, 378-392.

Cao, J., Spielmann, M., Qiu, X., Huang, X., Ibrahim, D.M., Hill, A.J., Zhang, F., Mundlos, S., Christiansen, L., Steemers, F.J., et al. (2019). The single-cell transcriptional landscape of mammalian organogenesis. Nature 566, 496-502.

Durante, M.A., Kurtenbach, S., Sargi, Z.B., Harbour, J.W., Choi, R., Kurtenbach, S., Goss, G.M., Matsunami, H., and Goldstein, B.J. (2020). Single-cell analysis of olfactory neurogenesis and differentiation in adult humans. Nat Neurosci.

Hansen, D.V., Lui, J.H., Parker, P.R., and Kriegstein, A.R. (2010). Neurogenic radial glia in the outer subventricular zone of human neocortex. Nature 464, 554-561.

Jin, K., Zhu, Y., Sun, Y., Mao, X.O., Xie, L., and Greenberg, D.A. (2002). Vascular endothelial growth factor (VEGF) stimulates neurogenesis in vitro and in vivo. Proceedings of the National Academy of Sciences of the United States of America 99, 11946-11950.

Kazanis, I., Lathia, J.D., Vadakkan, T.J., Raborn, E., Wan, R., Mughal, M.R., Eckley, D.M., Sasaki, T., Patton, B., Mattson, M.P., et al. (2010). Quiescence and activation of stem and precursor cell populations in the subependymal zone of the mammalian brain are associated with distinct cellular and extracellular matrix signals. The Journal of neuroscience : the official journal of the Society for Neuroscience 30, 9771-9781.

Kim, D.W., Yao, Z., Graybuck, L.T., Kim, T.K., Nguyen, T.N., Smith, K.A., Fong, O., Yi, L., Koulena, N., Pierson, N., et al. (2019). Multimodal Analysis of Cell Types in a Hypothalamic Node Controlling Social Behavior. Cell 179, 713-728 e717.

Leone, D.P., Heavner, W.E., Ferenczi, E.A., Dobreva, G., Huguenard, J.R., Grosschedl, R., and McConnell, S.K. (2015). Satb2 Regulates the Differentiation of Both Callosal and Subcerebral Projection Neurons in the Developing Cerebral Cortex. Cerebral cortex 25, 3406-3419.

Mancuso, R., Van Den Daele, J., Fattorelli, N., Wolfs, L., Balusu, S., Burton, O., Liston, A., Sierksma, A., Fourne, Y., Poovathingal, S., et al. (2019). Stem-cell-derived human microglia transplanted in mouse brain to study human disease. Nat Neurosci 22, 2111-2116.

Qiu, X., Hill, A., Packer, J., Lin, D., Ma, Y.A., and Trapnell, C. (2017). Single-cell mRNA quantification and differential analysis with Census. Nature methods 14, 309-315.

Rivera, C., Voipio, J., Payne, J.A., Ruusuvuori, E., Lahtinen, H., Lamsa, K., Pirvola, U., Saarma, M., and Kaila, K. (1999). The K+/Cl- co-transporter KCC2 renders GABA hyperpolarizing during neuronal maturation. Nature 397, 251-255.

Soliman, H., Paylor, B., Scott, R.W., Lemos, D.R., Chang, C., Arostegui, M., Low, M., Lee, C., Fiore, D., Braghetta, P., et al. (2020). Pathogenic Potential of Hic1-Expressing Cardiac Stromal Progenitors. Cell stem cell 26, 459-461.

Sun, Y.J., Jin, K.L., Childs, J.T., Xie, L., Mao, X.O., and Greenberg, D.A. (2006). Vascular endothelial growth factor-B (VEGFB) stimulates neurogenesis: Evidence from knockout mice and growth factor administration. Dev Biol 289, 329-335.

Trapnell, C., Cacchiarelli, D., Grimsby, J., Pokharel, P., Li, S., Morse, M., Lennon, N.J., Livak, K.J., Mikkelsen, T.S., and Rinn, J.L. (2014). The dynamics and regulators of cell fate decisions are revealed by pseudotemporal ordering of single cells. Nature biotechnology 32, 381-386.

Wang, X., Tsai, J.W., LaMonica, B., and Kriegstein, A.R. (2011). A new subtype of progenitor cell in the mouse embryonic neocortex. Nat Neurosci 14, 555-561.

Wen, S., Ma, D., Zhao, M., Xie, L., Wu, Q., Gou, L., Zhu, C., Fan, Y., Wang, H., and Yan, J. (2020). Spatiotemporal single-cell analysis of gene expression in the mouse suprachiasmatic nucleus. Nat Neurosci.

Wolf, F.A., Hamey, F.K., Plass, M., Solana, J., Dahlin, J.S., Gottgens, B., Rajewsky, N., Simon, L., and Theis, F.J. (2019). PAGA: graph abstraction reconciles clustering with trajectory inference through a topology preserving map of single cells. Genome biology 20, 59.

Yu, D.X., Di Giorgio, F.P., Yao, J., Marchetto, M.C., Brennand, K., Wright, R., Mei, A., Mchenry, L., Lisuk, D., Grasmick, J.M., et al. (2014). Modeling Hippocampal Neurogenesis Using Human Pluripotent Stem Cells. Stem cell reports 2, 295-310.
