# Supplementary material for: Vascularized human cortical organoids (vOrganoids) model cortical development in vivo
Source: PLoS Biol. 2020 May 13;18(5):e3000705. doi: 10.1371/journal.pbio.3000705 (PMC7250475; doi:10.1371/journal.pbio.3000705)
Supplement: Attachment — Submitted filename: Response.docx [file pbio.3000705.s014.docx]

MANUSCRIPT REQUIREMENTS

Please update your manuscript with the following changes:

1.	Please put all reference citations in square brackets, with no spaces, separated by either commas or dashes, e.g. [1,2,3] or [1-3].
**Response：All reference citations were cited in square brackets, with no spaces, separated by either commas or dashes.**

2.	Please make sure all references are cited in ascending numerical order in the text.
**Response：We have carefully checked all the references and ensured that they are cited in ascending numerical order in the text.**

3.	Please ensure that your article adheres to the standard layout and order of Abstract, Introduction, Results, Discussion, and (Materials and) Methods. Please do not place your (Materials and) Methods section after your References or your Supporting Information legends.
**Response：We have organized the article as follows: Title page with author list and author affiliations. The main text in an order of Abstract; Introduction; Results; Discussion; Materials and Methods; Acknowledgments; References; Supporting information.**

4.	Please ensure that all supporting information files which are intended for publication have the file type 'Supporting Information' in the file inventory. Your MDAR checklist currently has file type ‘Other’.
**Response：We have changed the file type of our MDAR checklist to “Supporting Information” and ensured that all supporting information files which are intended for publication have the file type ‘Supporting Information’ in the file inventory.**

FIGURE REQUIREMENTS

Please complete the following instructions regarding your figure files:

1.	Text in Figures 1, 2, and 3 appears pixelated/low quality. Please remake these parts of the figures using a setting such as "Smooth" in Photoshop or equivalent.
**Response：We have remaked the text in Figures 1,2 and 3 using a setting as “Smooth” in Photoshop.**

- Figure 3 has been submitted in a layered TIF file format, which we don’t accept for figures. To ensure that your figures publish properly, please flatten the layers in each figure file (Layer→Flatten Image in Photoshop/GIMP) and re-upload. Make sure to save your TIF files with LZW Compression turned on.

**Response：We have flatten the layers in Photoshop and saved with LZW compression..**

- Figure 4 is acceptable for publication.

You will find your submission in the 'Submissions Sent Back to Author' folder. Once you have made these changes, please re-approve the submission following the same steps as before.        

Please do not hesitate to contact me should you have any questions.

Best wishes,

Jessica Hyde
Publications Assistant
PLOS Biology

__________________________________________________
In compliance with data protection regulations, you may request that we remove your personal registration details at any time.  (Use the following URL: <https://www.editorialmanager.com/pbiology/login.asp?a=r>). Please contact the publication office if you have any questions.
